# Supplementary material for: Force Field Parameters for Fe2+4S2−4 Clusters of Dihydropyrimidine Dehydrogenase, the 5-Fluorouracil Cancer Drug Deactivation Protein: A Step towards In Silico Pharmacogenomics Studies
Source: Molecules. 2021 May 14;26(10):2929. doi: 10.3390/molecules26102929 (PMC8156676; doi:10.3390/molecules26102929)
Supplement: Supplementary file 1 [file molecules-26-02929-s001.zip › molecules-1210361-supplementary (1).pdf]

molecules

Supplementary Materials

Force Field Parameters for $Fe^{2+}{}_4S^{2-}{}_4$ Clusters of Dihydropyrimidine Dehydrogenase, the 5-Fluorouracil Cancer Drug Deactivation Protein: A Step towards In Silico Pharmacogenomics Studies

Maureen Bilinga Tendwa [1,†], Lorna Chebon-Bore [1,†], Kevin Lobb [1,2], Thommas Mutemi Musyoka [1,*] and Özlem Tastan Bishop [1,*]

[1] Research Unit in Bioinformatics (RUBi), Department of Biochemistry and Microbiology, Rhodes University, Makhanda 6140, South Africa; bilingatendwa@gmail.com (M.B.T.); lornajemosop@gmail.com (L.C.-B.); k.lobb@ru.ac.za (K.L.)
[2] Department of Chemistry, Rhodes University, Makhanda 6140, South Africa
* Correspondence: mutemibiochemistry@gmail.com (T.M.M.); o.tastanbishop@ru.ac.za (Ö.T.B.)
† These authors contributed equally to this work.

Table of Contents

Figure S1. An illustration Fe^{2+} center parameterization in the native human DPD Model 2, utilizing automated (VFFDT) Seminario approach.
Figure S2. An illustration of charge allocation to all the atoms coordinating with the metal center of subset clusters 1026A and 1027A for Model 1.
Figure S3. A representation of the root mean square of fluctuation (RMSF) for ligand bound (complex) and non-ligand bound (holo) DPD Model 1 during 150 ns simulation. **A)** $Fe^{2+}{}_4S^{2-}{}_4$ clusters in 1026 and 1027 located in domain 1. **B)** $Fe^{2+}{}_4S^{2-}{}_4$ clusters in 1028 and 1029 located in domain 5. The area of fluctuation coincides to the protein loop area while the iron cluster remains intact.
Figure S4. 3D structures of Model 2 MD simulations snapshots (timeframes) from regions exhibiting higher conformational changes with atomistic details represented **A**) at 110.0 ns for drug bound protein and **B)** at 70.4 ns for holo proteins (without 5-fluorouracil drug). The Fe^{2+} clusters remained intact throughout the different conformation timelines.
Figure S5: Color coded violin plots showing the crystal structure bond distances between the Fe^{2+} and S^{2-} derived by original (Model 1) and VFFDT [automated] (Model 2) Seminario method during 150 ns MD simulation. In Model 1, the orange and pink violin plots represent the holo and holo-drug bound complexes, respectively. In Model 2, the yellow and grey violin plots represent the holo and holo-drug complexes, respectively. The two clusters (1026 and 1027) represent the crystal structures (1H7X) unique $Fe^{2+}4S^{2-}4$ clusters coordination.
Table S1. Quality assessment of Human DPD protein modeled structures.
Table S2. Titratable residues in the human DPD protein and their respective pKa values.
Table S3. A representation of human DPD parameters and coordinate files for Model 1 (B3LYP/6-31G*): AMBER parameter file.
Table S4. A representation of human DPD parameters and coordinate files for Model 2 (LSDA/LANL2DZ): AMBER_VFFDT parameter file.
Table S5. Listing of charge allocation to all the atoms interacting with the metal center (B3LYP/6-31G*)
Table S6. Comparison of **A** bond length, **B** internal and **C** external angles (Å) calculated with X-ray, DFT (B3LYP) and (LSDA/LANL2DZ) method for the molecular cluster model ([$Fe^{2+}{}_4S^{2-}{}_4$ (S-Cys)$_3$(S-Gln)]) 1026A of Native DPD protein.
Table S7. Comparison of **A** bond length, **B** internal and **C** external angles (Å) calculated with X-ray, DFT (B3LYP) and (LSDA/LANL2DZ) method for the molecular cluster model ([Fe_4S_4(S-Cys)$_4$]) 1027A of native DPD protein.
Table S8. Dihedral related force constants for X-ray and post-MD simulation for both models' clusters ([Fe4S4(S-Cys)$_3$(S-Gln)]) and ([Fe_4S_4(S-Cys)$_4$]) of Native DPD protein.
Table S9. DPD *.pir* sequence file used for modeling human dihydropyramidine dehydrogenase structure based on pig crystal structure template and human target sequence

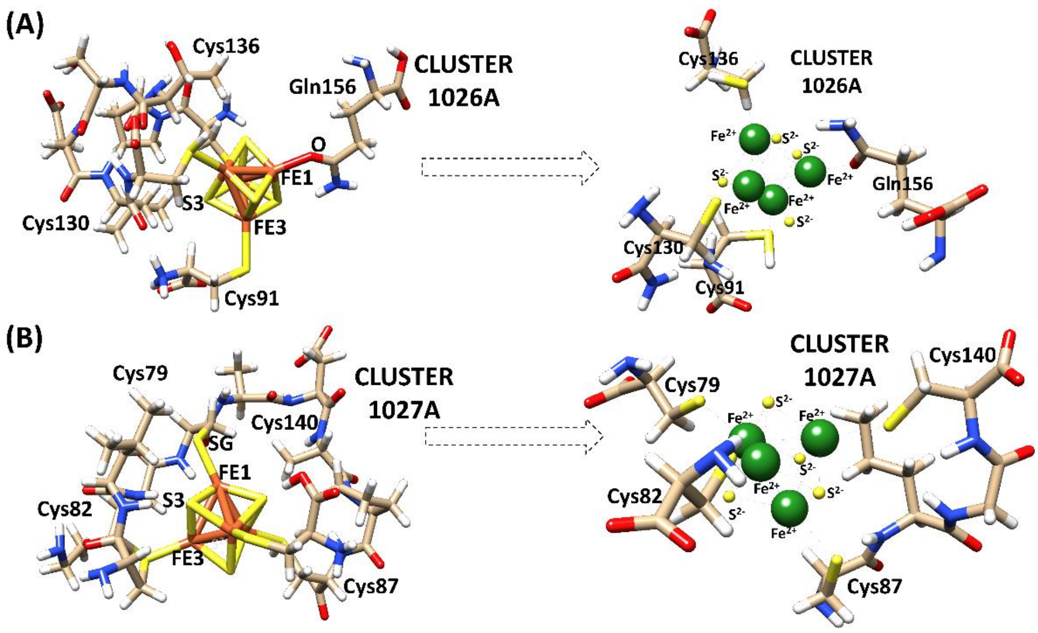

Figure S1. An illustration Fe^{2+} center parameterization in the native human DPD Model 2, utilizing automated (VFFDT) Seminario approach A**)** showing a 3D representation of coordinating Fe^{2+} sphere for cluster 1026A and an adjacent structure which has undergone parameterization, similarly **B)** shows a 3D representation of coordinating Fe^{2+} sphere for cluster 1027A and an adjacent structure which has undergone parameterization.

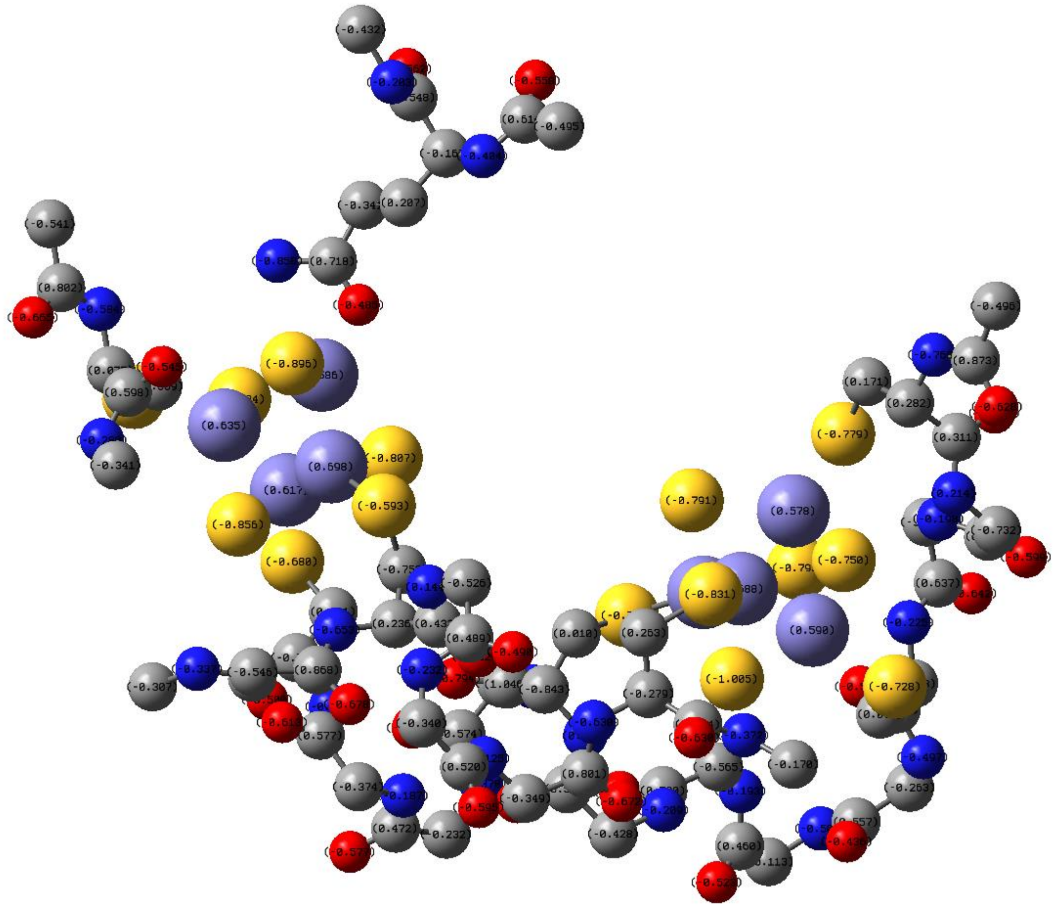

Figure S2. An illustration of charge allocation to all the atoms coordinating with the metal center of subset clusters 1026A and 1027A for Model 1**.** Purple, yellow, red, blue, and grey spheres represent Iron, sulfur, oxygen, nitrogen, and carbon atoms, respectively.

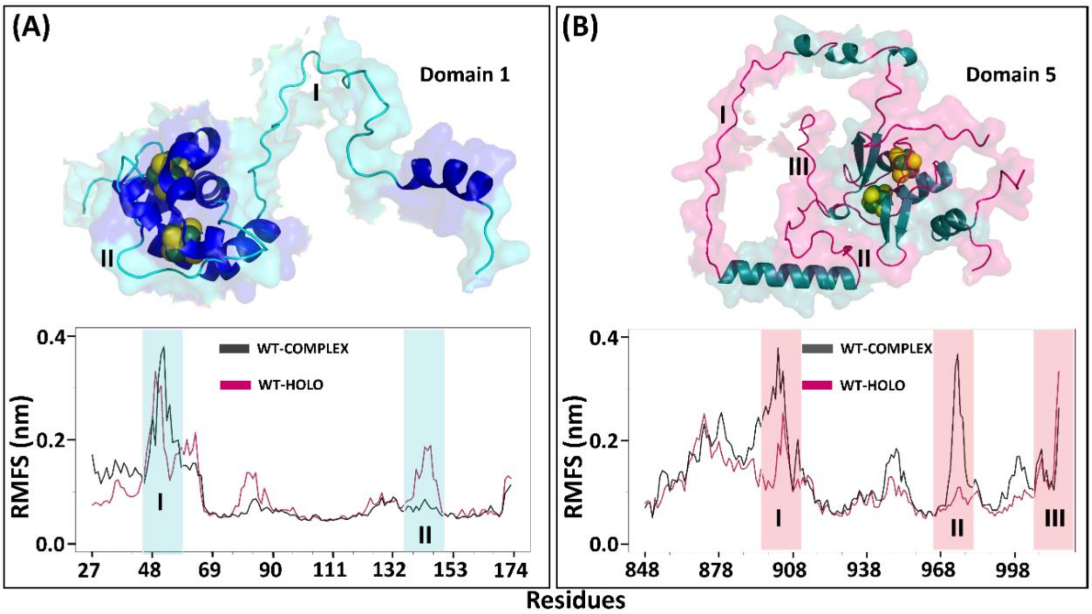

Figure S3. A representation of the root mean square of fluctuation (RMSF) for ligand bound (complex) and non-ligand bound (holo) DPD Model 1 during 150 ns simulation**. A)** $Fe^{2+}{}_4S^{2-}{}_4$ clusters in 1026 and 1027 located in domain 1. **B)** $Fe^{2+}{}_4S^{2-}{}_4$ clusters in 1028 and 1029 located in domain 5. The area of fluctuation coincides to the protein loop area while the iron cluster remains intact.

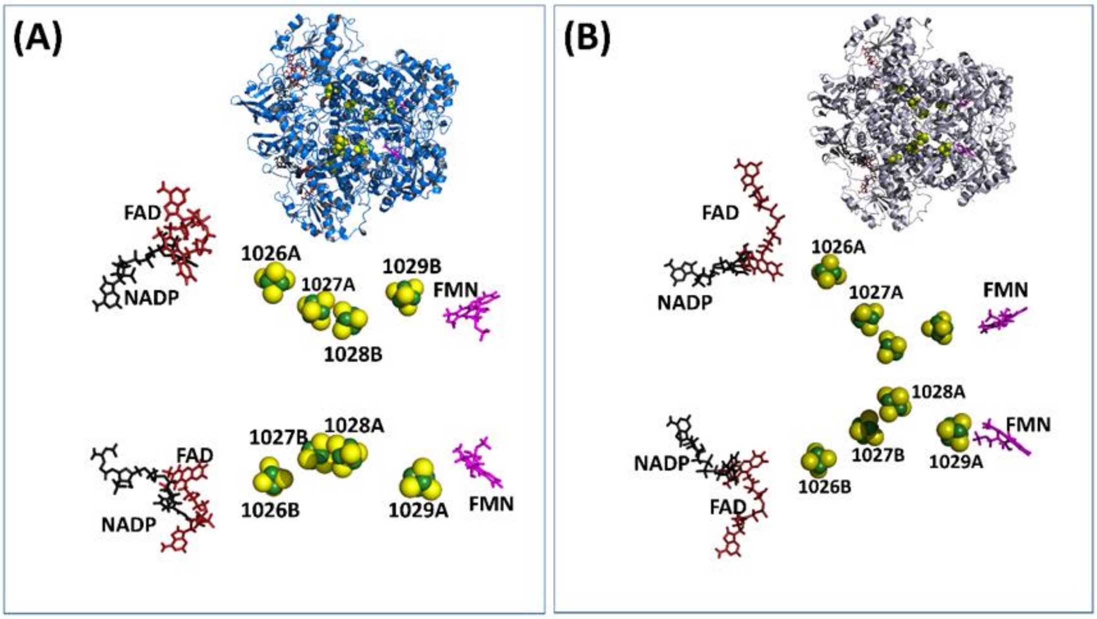

Figure S4. 3D structures of Model 2 MD simulations snapshots (timeframes) from regions exhibiting higher conformational changes with atomistic details represented **A**) at 110.0 ns for drug bound protein and **B)** at 70.4 ns for holo proteins (without 5-fluorouracil drug). The Fe^{2+} clusters remained intact throughout the different conformation timelines.

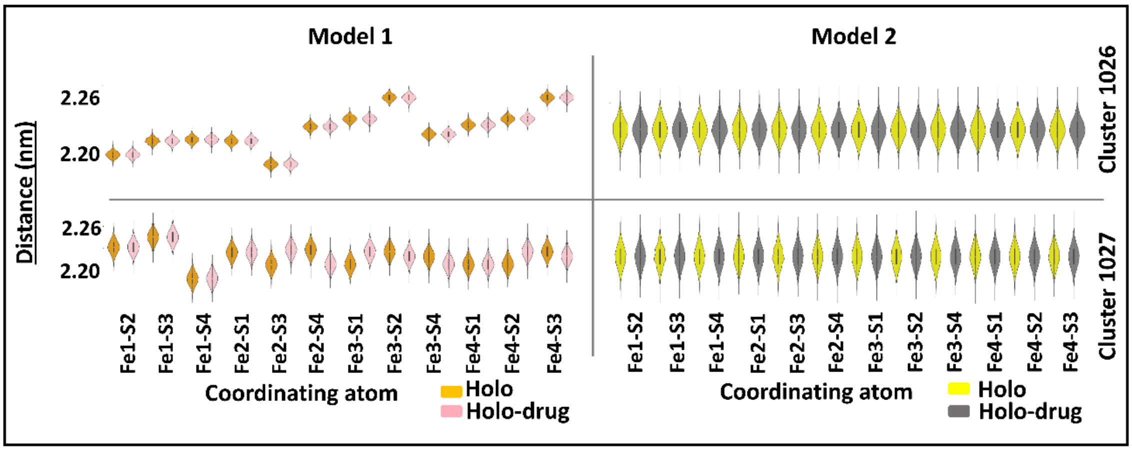

Figure S5: Color coded violin plots showing the crystal structure bond distances between the Fe^{2+} and S^{2-} derived by original (Model 1) and VFFDT [automated] (Model 2) Seminario method during 150 ns MD simulation. In Model 1, the orange and pink violin plots represent the holo and holo-drug bound complexes, respectively. In Model 2, the yellow and grey violin plots represent the holo and holo-drug complexes, respectively. The two clusters (1026 and 1027) represent the crystal structures (1H7X) unique $Fe^{2+}4S^{2-}4$ clusters coordination.

Table S1: Quality assessment of Human DPD protein modeled structures before and after 150 ns molecular dynamic simulation

		Pre and Post MD human DPD protein model structure quality validation						
**PROTEINS**		VERIFY 3D (%)	**QMEAN6**	**ProSA**		**PROCHECK (%)**		
		**3D-ID score**	**QMEAN score**	**Monomers Z-score**		**Ramachandran (residues location)**		
				**A**	**B**	**Favored**	**Allowed**	**Disallowed**
Template	Pre-MD	85.79	0.91	-13.56	-13.47	93.5	6.4	0.1
	Post-MD	84.30	0.84	-12.2	-12.53	84.4	15.1	0.0
Holo	Pre-MD	85.01	0.90	-13.41	-13.44	89.4	10.2	0.0
	Post-MD	82.46	0.83	-12.22	-12.17	83.8	16.0	0.2
Holo-drug	Pre-MD	85.01	0.89	- 13.42	-13.45	92.2	7.7	0.1
	Post-MD	81.92	0.83	- 12.61	-12.62	85.8	14.1	0.1

MD: Molecular dynamics simulations

Table S2. Titratable residues in the human DPD protein and their respective pKa values

Residue	pK (int)	pKa_(1/2)	Residue	pK (int)	pKa_(1/2)	Residue	pK (int)	pKa_(1/2)
**Chain-A**								
NTALA-2	7.417	7.972	ARG-678	11.484	>12.000	CYS-324	8.990	9.066
LYS-7	10.092	11.678	CYS-684	11.950	>12.000	HIS-325	6.186	6.037
ASP-8	4.515	0.918	ASP-687	3.873	1.358	ARG-332	12.252	>12.000
ASP-10	3.600	3.978	GLU-689	4.008	3.203	ASP-342	2.523	<0.000
ASP-11	3.436	3.435	ARG-692	11.329	>12.000	ASP-346	4.452	<0.000
GLU-13	4.390	4.841	CYS-695	11.905	>12.000	CYS-347	9.618	>12.000
ARG-21	11.752	>12.000	ARG-696	11.889	>12.000	ARG-353	12.006	>12.000
HIS-25	6.649	7.179	ARG-699	12.639	>12.000	CYS-354	12.003	>12.000
CYS-29	9.529	10.217	LYS-709	6.035	11.023	ARG-357	11.430	>12.000
LYS-34	9.536	>12.000	ASP-716	2.681	<0.000	ARG-358	11.391	>12.000
LYS-35	9.563	>12.000	ARG-722	11.424	>12.000	ARG-364	11.276	>12.000
ASP-37	5.142	1.200	LYS-725	10.304	10.679	LYS-365	9.534	9.163
LYS-38	9.329	>12.000	GLU-726	5.068	2.383	ARG-371	11.195	>12.000
LYS-39	10.143	10.937	ASP-730	5.918	2.792	GLU-375	3.112	<0.000
HIS-40	6.461	6.871	LYS-745	9.516	>12.000	GLU-376	4.400	6.860
LYS-42	9.998	>12.000	ASP-747	3.896	<0.000	GLU-378	4.413	<0.000
ARG-43	13.175	>12.000	LYS-758	10.327	11.421	LYS-381	8.938	>12.000
ASP-46	4.116	<0.000	ARG-759	12.228	>12.000	GLU-382	5.675	<0.000
LYS-47	9.719	>12.000	TYR-762	14.594	>12.000	GLU-383	6.836	<0.000
CYS-49	9.248	9.348	ARG-771	10.083	>12.000	LYS-384	10.693	>12.000
CYS-52	10.767	>12.000	ARG-776	10.282	>12.000	CYS-385	11.195	>12.000
GLU-53	5.888	0.730	ARG-783	11.892	>12.000	GLU-386	5.553	<0.000
LYS-54	9.683	10.896	ASP-797	4.647	<0.000	ARG-394	11.524	>12.000
GLU-56	6.258	<0.000	GLU-800	3.816	<0.000	LYS-395	10.171	10.585
ASP-60	5.726	<0.000	HIS-807	5.207	3.368	LYS-399	10.108	10.435
ASP-61	5.047	<0.000	CYS-816	7.570	7.492	ARG-402	12.151	>12.000
LYS-63	9.179	>12.000	ASP-823	2.599	<0.000	ARG-410	11.826	>12.000

HIS-64 5.720 9.050	GLU-828 3.751 5.528	GLU-412 5.136 0.902
GLU-69 3.222 <0.000	ASP-829 1.478 <0.000	ASP-414 3.839 2.274
ARG-70 8.644 >12.000	TYR-830 10.911 >12.000	GLU-415 4.126 3.367
ARG-74 10.915 >12.000	CYS-831 9.358 >12.000	LYS-418 10.218 10.910
GLU-75 4.124 <0.000	LYS-835 9.380 >12.000	GLU-421 4.361 1.315
ARG-78 10.937 >12.000	TYR-839 12.375 >12.000	ASP-422 4.847 5.473
CYS-79 7.155 8.371	LYS-841 9.611 >12.000	GLU-423 5.357 3.329
LYS-81 9.191 >12.000	GLU-844 4.631 3.112	ASP-424 3.742 2.658
CYS-82 6.821 >12.000	GLU-845 5.283 5.206	HID-428 6.245 6.107
ASP-84 5.617 <0.000	ASP-848 4.229 3.553	LYS-430 9.983 >12.000
CYS-87 5.793 >12.000	ASP-850 3.751 <0.000	ASP-432 4.667 2.550
LYS-89 10.120 >12.000	HIS-859 2.394 <0.000	ASP-444 2.973 1.331
CYS-91 6.640 8.917	LYS-861 10.276 >12.000	LYS-446 9.801 10.964
HIS-94 5.713 0.409	LYS-863 10.097 >12.000	LYS-448 9.760 11.020
ASP-96 2.303 <0.000	ARG-867 11.666 >12.000	GLU-449 4.386 4.021
LYS-98 6.466 >12.000	GLU-870 6.530 3.817	LYS-455 10.222 11.077
LYS-107 8.251 >12.000	ASP-873 3.827 3.252	ARG-458 11.797 >12.000
TYR-109 12.293 >12.000	LYS-874 10.222 11.604	GLU-463 4.330 4.038
TYR-110 11.905 >12.000	LYS-875 10.158 >12.000	ASP-465 3.138 2.845
LYS-114 8.864 >12.000	TYR-882 11.326 >12.000	GLU-467 3.882 3.682
ASP-119 5.422 <0.000	GLU-884 4.874 2.698	GLU-473 4.164 3.200
CYS-126 6.601 >12.000	ARG-886 11.628 >12.000	ASP-481 1.702 <0.000
CYS-130 6.202 >12.000	LYS-887 10.151 >12.000	GLU-491 3.011 <0.000
ASP-134 4.533 <0.000	LYS-888 10.369 11.315	ASP-495 2.170 <0.000
CYS-136 6.036 >12.000	GLU-892 5.494 4.079	LYS-497 8.758 >12.000
CYS-140 6.456 5.546	GLU-893 5.290 3.716	TYR-502 10.836 >12.000
TYR-143 10.672 >12.000	LYS-894 9.690 >12.000	HIS-504 5.309 4.651
GLU-146 4.597 <0.000	ARG-896 12.114 >12.000	LYS-505 10.092 10.876
GLU-147 5.224 <0.000	LYS-898 10.086 >12.000	TYR-506 11.696 >12.000
GLU-161 4.307 2.857	GLU-899 4.934 3.926	TYR-511 11.474 >12.000
LYS-164 9.536 >12.000	LYS-908 10.362 10.775	LYS-518 9.969 >12.000

ARG-172 13.132 >12.000	ARG-909 11.520 >12.000	GLU-520 5.117 2.691
GLU-180 4.415 3.790	CYS-911 10.130 11.404	TYR-525 11.106 >12.000
LYS-181 10.258 10.573	LYS-915 10.243 11.792	ASP-529 4.834 <0.000
GLU-184 4.462 3.974	ARG-916 11.939 >12.000	ASP-532 3.498 2.437
TYR-186 11.038 11.742	LYS-922 10.179 11.304	GLU-536 4.535 3.981
LYS-189 10.624 >12.000	ASP-923 4.440 2.744	LYS-541 10.366 11.337
CYS-202 10.875 >12.000	LYS-927 10.571 10.938	ARG-561 11.833 >12.000
ARG-208 11.892 >12.000	TYR-931 11.367 >12.000	ARG-562 9.217 >12.000
TYR-211 12.788 >12.000	GLU-937 4.620 2.157	GLU-565 4.518 3.413
ASP-213 4.946 1.769	GLU-942 4.228 3.649	LYS-574 7.509 >12.000
GLU-218 2.317 <0.000	ASP-949 3.028 0.531	ASP-579 3.747 2.915
LYS-219 8.693 >12.000	GLU-950 4.668 5.855	LYS-580 9.498 >12.000
GLU-221 4.132 3.561	GLU-951 5.702 1.520	ASP-581 4.111 3.531
TYR-222 9.094 >12.000	CYS-953 7.496 >12.000	ARG-589 8.869 >12.000
GLU-230 3.569 <0.000	CYS-956 7.107 1.932	ARG-592 12.314 >12.000
ARG-235 9.353 >12.000	LYS-958 7.074 >12.000	TYR-600 10.708 >12.000
TYR-238 11.091 >12.000	CYS-959 6.653 <0.000	GLU-611 5.412 <0.000
ASP-239 4.261 0.642	TYR-960 12.121 >12.000	GLU-615 4.869 <0.000
GLU-244 2.724 <0.000	CYS-963 5.350 11.686	LYS-616 8.766 >12.000
GLU-246 4.835 3.403	ASP-965 2.428 <0.000	TYR-620 10.801 >12.000
LYS-249 9.374 >12.000	TYR-968 11.555 >12.000	CYS-622 11.760 >12.000
ASP-250 4.804 3.488	ASP-974 2.649 0.129	GLU-627 5.247 3.649
LYS-254 10.056 >12.000	GLU-976 4.583 3.605	LYS-629 10.732 >12.000
CYS-257 9.490 >12.000	HIS-978 5.305 9.984	ASP-631 5.100 2.340
LYS-259 10.095 10.908	ASP-984 4.401 3.121	ASP-634 4.392 3.873
GLU-265 4.494 3.918	CYS-986 7.289 >12.000	CYS-643 10.096 >12.000
LYS-272 9.978 11.037	CYS-989 6.958 8.920	TYR-645 10.610 >12.000
GLU-273 4.767 3.907	CYS-992 5.732 <0.000	LYS-647 10.186 10.183
LYS-274 9.903 10.056	CYS-996 7.455 10.503	ASP-649 4.813 1.835
TYR-276 11.567 >12.000	ASP-1000 3.607 <0.000	GLU-652 4.490 4.296
LYS-277 10.604 11.424	CYS-1001 8.150 >12.000	LYS-655 10.192 11.038

GLU-287 3.240 2.792	LYS-1002 3.228 >12.000	LYS-656 9.621 >12.000
LYS-290 9.997 11.006	ARG-1003 10.588 >12.000	GLU-658 5.018 4.231
ASP-291 3.660 1.830	ARG-1007 11.908 >12.000	ASP-659 6.916 7.080
ASP-300 4.078 3.428	TYR-1011 12.126 >12.000	ASP-663 5.385 1.746
TYR-304 11.589 >12.000	GLU-1012 4.464 3.817	GLU-666 4.003 <0.000
LYS-307 7.682 >12.000	LYS-1014 9.453 11.036	CYS-671 9.081 10.646
ASP-308 2.913 1.506	ARG-1015 12.068 >12.000	HIS-673 4.876 5.868
LYS-315 9.571 >12.000	LEU-1017 3.812 2.839	GLU-677 4.172 3.721
LYS-318 10.523 >12.000	**Chain-B**	ARG-678 11.497 >12.000
CYS-322 8.439 8.884	NTALA-2 7.303 7.567	CYS-684 10.768 >12.000
CYS-324 8.474 9.151	LYS-7 10.092 11.514	ASP-687 3.422 1.126
HIS-325 6.353 6.228	ASP-8 4.485 0.829	GLU-689 3.988 3.859
ARG-332 12.495 >12.000	ASP-11 3.481 3.217	ARG-692 11.261 >12.000
ASP-342 2.251 <0.000	GLU-13 4.381 4.529	CYS-695 11.885 >12.000
ASP-346 3.570 <0.000	ARG-21 11.916 >12.000	ARG-696 11.809 >12.000
CYS-347 9.395 >12.000	HIS-25 6.433 7.127	ARG-699 13.391 >12.000
ARG-353 12.182 >12.000	CYS-29 9.514 10.390	LYS-709 6.259 >12.000
CYS-354 12.357 >12.000	LYS-34 9.319 >12.000	ASP-716 3.023 <0.000
ARG-357 11.291 >12.000	LYS-35 8.741 10.536	ARG-722 11.522 >12.000
ARG-358 10.450 >12.000	ASP-37 4.901 0.807	LYS-725 10.246 10.437
ARG-364 11.344 >12.000	LYS-38 8.850 >12.000	GLU-726 5.090 2.468
LYS-365 9.444 8.767	LYS-39 10.086 11.352	LYS-745 9.406 >12.000
ARG-371 11.444 >12.000	HIS-40 6.881 7.522	ASP-747 4.039 <0.000
GLU-375 3.182 <0.000	LYS-42 9.927 >12.000	LYS-758 10.182 11.247
GLU-376 4.609 7.909	ARG-43 13.227 >12.000	ARG-759 12.513 >12.000
GLU-378 4.472 <0.000	ASP-46 3.757 <0.000	TYR-762 13.151 >12.000
LYS-381 8.961 >12.000	LYS-47 9.620 >12.000	ARG-771 9.652 >12.000
GLU-382 5.890 <0.000	CYS-49 9.602 9.668	ARG-776 10.476 >12.000
GLU-383 7.131 2.331	CYS-52 9.631 10.695	ARG-783 12.031 >12.000
LYS-384 10.273 >12.000	GLU-53 5.615 1.985	ASP-797 4.369 <0.000
CYS-385 11.369 >12.000	LYS-54 10.179 >12.000	GLU-800 3.723 <0.000

GLU-386	5.498	<0.000	GLU-56	6.027	0.376	HIS-807	4.995	2.920
ARG-394	12.049	>12.000	ASP-60	5.580	<0.000	CYS-816	7.583	7.301
LYS-395	10.185	10.533	ASP-61	4.931	<0.000	ASP-823	2.425	<0.000
LYS-399	10.127	10.237	LYS-63	9.065	>12.000	GLU-828	3.787	5.495
ARG-402	12.348	>12.000	HIS-64	5.372	8.066	ASP-829	1.085	<0.000
ARG-410	12.126	>12.000	GLU-69	3.049	<0.000	TYR-830	10.028	>12.000
GLU-412	5.336	3.049	ARG-70	8.610	>12.000	CYS-831	9.409	>12.000
ASP-414	3.957	2.361	ARG-74	10.614	>12.000	LYS-835	9.531	>12.000
GLU-415	4.611	3.124	GLU-75	4.176	<0.000	TYR-839	12.083	>12.000
LYS-418	10.487	11.457	ARG-78	11.185	>12.000	LYS-841	9.345	>12.000
GLU-421	5.204	2.049	CYS-79	7.452	10.201	GLU-844	4.858	3.424
ASP-422	5.009	6.599	LYS-81	9.384	>12.000	GLU-845	4.934	4.976
GLU-423	4.637	2.362	CYS-82	6.829	>12.000	ASP-848	4.196	3.298
ASP-424	3.906	3.001	ASP-84	5.188	<0.000	ASP-850	3.373	<0.000
HID-428	6.190	5.720	CYS-87	6.049	>12.000	HIS-859	4.635	5.008
LYS-430	10.049	11.656	LYS-89	10.015	>12.000	LYS-861	10.211	>12.000
ASP-432	4.726	2.443	CYS-91	6.684	9.325	LYS-863	9.993	>12.000
ASP-444	3.025	1.501	HIS-94	5.457	<0.000	ARG-867	11.714	>12.000
LYS-446	9.783	10.975	ASP-96	1.971	<0.000	GLU-870	5.014	3.574
LYS-448	10.003	11.215	LYS-98	6.142	>12.000	ASP-873	3.845	3.093
GLU-449	4.446	4.084	LYS-107	8.064	>12.000	LYS-874	10.169	11.356
LYS-455	10.237	11.121	TYR-109	11.951	>12.000	LYS-875	10.049	>12.000
ARG-458	11.879	>12.000	TYR-110	12.459	>12.000	TYR-882	11.538	>12.000
GLU-463	4.395	4.226	LYS-114	8.918	>12.000	GLU-884	4.766	2.427
ASP-465	2.478	1.902	ASP-119	5.577	<0.000	ARG-886	11.545	>12.000
GLU-467	3.620	3.475	CYS-126	7.598	>12.000	LYS-887	10.081	>12.000
GLU-473	4.169	3.252	CYS-130	6.616	>12.000	LYS-888	10.359	11.369
ASP-481	1.697	<0.000	ASP-134	4.849	<0.000	GLU-892	5.120	3.584
GLU-491	1.527	<0.000	CYS-136	5.948	>12.000	LYS-894	9.588	>12.000
ASP-495	1.865	<0.000	CYS-140	6.569	6.413	ARG-896	12.028	>12.000
LYS-497	8.910	>12.000	TYR-143	11.062	>12.000	LYS-898	10.313	>12.000

TYR-502	10.721	>12.000	GLU-146	4.899	<0.000	GLU-899	4.713	3.870
HIS-504	5.529	5.057	GLU-147	5.091	<0.000	GLU-908	4.451	4.161
LYS-505	10.020	10.886	GLU-161	3.736	<0.000	ARG-909	12.053	>12.000
TYR-506	11.472	>12.000	LYS-164	8.930	>12.000	ASP-910	4.599	4.392
TYR-511	11.391	>12.000	ARG-172	12.886	>12.000	CYS-911	11.082	>12.000
LYS-518	10.405	>12.000	GLU-180	4.340	3.833	LYS-915	10.368	>12.000
GLU-520	4.694	2.460	LYS-181	10.263	10.540	ARG-916	11.973	>12.000
TYR-525	11.171	>12.000	GLU-184	4.454	3.989	LYS-922	10.215	11.315
ASP-529	4.550	<0.000	TYR-186	10.640	11.045	ASP-923	3.488	1.495
ASP-532	3.367	2.155	LYS-189	10.554	11.622	LYS-927	10.526	10.847
GLU-536	4.520	4.070	CYS-202	11.328	>12.000	TYR-931	11.357	>12.000
LYS-541	10.375	11.439	ARG-208	12.173	>12.000	GLU-937	5.376	3.135
ARG-561	11.682	>12.000	TYR-211	14.406	>12.000	GLU-942	4.193	3.579
ARG-562	9.172	>12.000	ASP-213	5.427	2.277	ASP-949	3.081	0.434
GLU-565	4.588	3.426	GLU-218	2.676	<0.000	GLU-950	4.768	5.720
LYS-574	7.621	>12.000	LYS-219	8.580	>12.000	GLU-951	5.644	0.188
ASP-579	5.227	9.491	GLU-221	4.261	3.937	CYS-953	7.489	>12.000
LYS-580	9.587	>12.000	TYR-222	9.707	>12.000	CYS-956	7.052	<0.000
ASP-581	4.074	3.498	GLU-230	4.551	0.334	LYS-958	7.356	>12.000
ARG-589	8.773	>12.000	ARG-235	9.374	>12.000	CYS-959	7.047	<0.000
ARG-592	12.279	>12.000	TYR-238	11.339	>12.000	TYR-960	12.823	>12.000
TYR-600	10.478	>12.000	ASP-239	3.611	<0.000	CYS-963	5.987	>12.000
GLU-611	4.902	<0.000	GLU-244	3.536	<0.000	ASP-965	2.615	<0.000
GLU-615	4.875	<0.000	GLU-246	5.141	4.394	TYR-968	11.129	>12.000
LYS-616	8.724	>12.000	LYS-249	9.644	>12.000	ASP-974	3.421	1.243
TYR-620	11.923	>12.000	ASP-250	5.261	4.174	GLU-976	4.826	3.810
CYS-622	11.465	>12.000	LYS-254	10.513	>12.000	HIS-978	5.660	10.280
GLU-627	3.900	1.193	CYS-257	9.804	>12.000	ASP-984	4.239	2.667
LYS-629	10.761	>12.000	LYS-259	10.095	10.949	CYS-986	7.143	9.614
ASP-631	4.956	2.740	GLU-265	4.239	3.526	CYS-989	7.164	6.202
ASP-634	4.364	3.885	LYS-272	10.041	11.198	CYS-992	6.714	>12.000

CYS-643	9.773	>12.000	GLU-273	4.637	3.923	CYS-996	7.589	9.756
TYR-645	10.854	11.797	LYS-274	9.928	9.807	ASP-1000	3.752	<0.000
LYS-647	9.652	9.712	TYR-276	11.844	>12.000	CYS-1001	8.191	>12.000
ASP-649	4.764	2.051	LYS-277	10.656	11.399	LYS-1002	4.736	>12.000
GLU-652	4.299	4.117	GLU-287	3.373	3.024	ARG-1003	10.742	>12.000
LYS-655	9.934	10.986	LYS-290	9.694	10.778	ARG-1007	11.792	>12.000
LYS-656	9.203	11.476	ASP-291	3.366	1.735	TYR-1011	12.215	>12.000
GLU-658	4.790	4.743	ASP-300	3.895	3.517	GLU-1012	4.463	3.777
ASP-659	4.923	4.970	TYR-304	11.644	>12.000	LYS-1014	9.489	10.840
ASP-663	5.380	1.857	LYS-307	7.604	>12.000	ARG-1015	11.964	>12.000
GLU-666	3.471	<0.000	ASP-308	2.671	1.112	CTala-2035	4.036	2.880
CYS-671	10.614	>12.000	LYS-315	9.386	>12.000			
HIS-673	5.824	6.323	LYS-318	11.015	>12.000			
GLU-677	4.057	2.963	CYS-322	9.919	11.243			

Table S3. A representation of human DPD parameters and coordinate files for Model 1 (B3LYP/6-31G*): AMBER parameter file

##Model 1 (B3LYP/6-31G*) AMBER parameter file

REMARK GOES HERE, THIS FILE IS GENERATED BY MCPB.PY

MASS			
FE1	55.85		Fe ion
FE2	55.85		Fe ion
FE3	55.85		Fe ion
FE4	55.85		Fe ion
FE5	55.85		Fe ion
FE6	55.85		Fe ion
FE7	55.85		Fe ion
FE8	55.85		Fe ion
CysSG(78)	32.06	2.900	S in cystine
CysSG(81)	32.06	2.900	S in cystine
CysSG(86)	32.06	2.900	S in cystine
CysSG(90)	32.06	2.900	S in cystine
CysSG(129)	32.06	2.900	S in cystine
CysSG(135)	32.06	2.900	S in cystine
CysSG(139)	32.06	2.900	S in cystine
GnOE(155)	16.00	0.434	carbonyl group oxygen
S1	32.060	2.900	S ion
S2	32.060	2.900	S ion
S3	32.060	2.900	S ion
S4	32.060	2.900	S ion
S5	32.060	2.900	S ion
S6	32.060	2.900	S ion
S7	32.060	2.900	S ion
S8	32.060	2.900	S ion

BOND	(kcal/mol/Å)	(Å)	
CysSG(90)-FE1	44.6	2.3851	Created by Seminario method using MCPB.py
S2-FE1	56.1	2.2759	Created by Seminario method using MCPB.py
S2-FE3	57.3	2.3031	Created by Seminario method using MCPB.py
S2-FE4	54.0	2.2876	Created by Seminario method using MCPB.py
S3-FE1	55.1	2.2007	Created by Seminario method using MCPB.py
S3-FE2	66.5	2.1837	Created by Seminario method using MCPB.py
S3-FE4	55.6	2.1893	Created by Seminario method using MCPB.py
S4-FE1	66.1	2.2277	Created by Seminario method using MCPB.py
S4-FE2	62.0	2.2510	Created by Seminario method using MCPB.py
S4-FE3	76.3	2.2375	Created by Seminario method using MCPB.py
CysSG(135)-FE2	53.4	2.3262	Created by Seminario method using MCPB.py
S1-FE2	54.7	2.2552	Created by Seminario method using MCPB.py
S1-FE3	49.3	2.2649	Created by Seminario method using MCPB.py
S1-FE4	50.5	2.2541	Created by Seminario method using MCPB.py
GnOE(155)-FE3	60.4	1.9176	Created by Seminario method using MCPB.py
CysSG(129)-FE4	36.4	2.4150	Created by Seminario method using MCPB.py
CysSG(139)-FE5	36.4	2.3965	Created by Seminario method using MCPB.py
S6-FE5	45.1	2.2410	Created by Seminario method using MCPB.py
S6-FE7	45.3	2.2325	Created by Seminario method using MCPB.py
S6-FE8	41.5	2.2698	Created by Seminario method using MCPB.py

S7-FE5	64.3	2.2502	Created by Seminario method using MCPB.py
S7-FE6	69.0	2.2387	Created by Seminario method using MCPB.py
S7-FE8	61.8	2.2645	Created by Seminario method using MCPB.py
S8-FE5	58.3	2.2124	Created by Seminario method using MCPB.py
S8-FE6	51.2	2.2250	Created by Seminario method using MCPB.py
S8-FE7	64.7	2.1906	Created by Seminario method using MCPB.py
CysSG(86)-FE6	50.0	2.3515	Created by Seminario method using MCPB.py
S5-FE6	66.3	2.2363	Created by Seminario method using MCPB.py
S5-FE7	60.8	2.2371	Created by Seminario method using MCPB.py
S5-FE8	57.0	2.2642	Created by Seminario method using MCPB.py
CysSG(78)-FE7	40.2	2.3710	Created by Seminario method using MCPB.py
CysSG(81)-FE8	37.1	2.4103	Created by Seminario method using MCPB.py
C -GnOE(155)	570.0	1.229	JCC,7,(1986),230; AA,CYT,GUA,THY,URA
CT-CysSG(90)	237.0	1.810	changed from 222.0 based on methanethiol nmodes
CT-CysSG(135)	237.0	1.810	changed from 222.0 based on methanethiol nmodes
CT-CysSG(129)	237.0	1.810	changed from 222.0 based on methanethiol nmodes
CT-CysSG(139)	237.0	1.810	changed from 222.0 based on methanethiol nmodes
CT-CysSG(86)	237.0	1.810	changed from 222.0 based on methanethiol nmodes
CT-CysSG(78)	237.0	1.810	changed from 222.0 based on methanethiol nmodes
CT-CysSG(81)	237.0	1.810	changed from 222.0 based on methanethiol nmodes

ANGLE	(kcal mol/rad)	(degrees)	
C -GnOE(155)-FE3	75.86	130.30	Created by Seminario method using MCPB.py
CT-CysSG(90)-FE1	106.68	103.98	Created by Seminario method using MCPB.py
CT-CysSG(135)-FE2	100.90	107.39	Created by Seminario method using MCPB.py
CT-CysSG(129)-FE4	97.96	96.66	Created by Seminario method using MCPB.py
CT-CysSG(139)-FE5	94.60	105.21	Created by Seminario method using MCPB.py
CT-CysSG(86)-FE6	101.58	107.61	Created by Seminario method using MCPB.py
CT-CysSG(78)-FE7	100.02	108.65	Created by Seminario method using MCPB.py
CT-CysSG(81)-FE8	106.65	103.60	Created by Seminario method using MCPB.py
FE2-S3-FE1	75.11	68.39	Created by Seminario method using MCPB.py
FE2-S4-FE1	80.79	66.76	Created by Seminario method using MCPB.py
FE3-S2-FE1	23.20	69.98	Created by Seminario method using MCPB.py
FE3-S4-FE1	26.76	72.04	Created by Seminario method using MCPB.py
FE3-S4-FE2	40.49	66.60	Created by Seminario method using MCPB.py
FE3-S1-FE2	47.73	66.08	Created by Seminario method using MCPB.py
FE4-S2-FE1	53.67	65.65	Created by Seminario method using MCPB.py
FE4-S2-FE3	20.95	64.15	Created by Seminario method using MCPB.py
FE4-S3-FE1	29.62	68.59	Created by Seminario method using MCPB.py
FE4-S3-FE2	61.07	68.33	Created by Seminario method using MCPB.py
FE4-S1-FE2	78.37	66.00	Created by Seminario method using MCPB.py
FE4-S1-FE3	53.87	65.30	Created by Seminario method using MCPB.py
FE6-S7-FE5	63.39	66.89	Created by Seminario method using MCPB.py
FE6-S8-FE5	77.31	67.77	Created by Seminario method using MCPB.py
FE7-S6-FE5	42.52	67.16	Created by Seminario method using MCPB.py
FE7-S8-FE5	64.00	68.38	Created by Seminario method using MCPB.py
FE7-S8-FE6	42.93	68.59	Created by Seminario method using MCPB.py
FE7-S5-FE6	35.00	67.59	Created by Seminario method using MCPB.py
FE8-S6-FE5	61.00	67.08	Created by Seminario method using MCPB.py
FE8-S6-FE7	44.11	66.60	Created by Seminario method using MCPB.py
FE8-S7-FE5	46.02	67.02	Created by Seminario method using MCPB.py
FE8-S7-FE6	47.60	70.85	Created by Seminario method using MCPB.py
FE8-S5-FE6	36.63	70.89	Created by Seminario method using MCPB.py
FE8-S5-FE7	67.61	66.62	Created by Seminario method using MCPB.py
CysSG(90)-FE1-S2	41.30	111.43	Created by Seminario method using MCPB.py

CysSG(90)-FE1-S3	63.82	115.56	Created by Seminario method using MCPB.py
CysSG(90)-FE1-S4	65.80	106.49	Created by Seminario method using MCPB.py
S2-FE1-S3	43.74	109.45	Created by Seminario method using MCPB.py
S2-FE1-S4	28.56	103.16	Created by Seminario method using MCPB.py
S2-FE3-S4	28.52	101.98	Created by Seminario method using MCPB.py
S2-FE4-S3	60.52	109.42	Created by Seminario method using MCPB.py
S3-FE1-S4	35.95	109.98	Created by Seminario method using MCPB.py
S3-FE2-S4	69.35	109.74	Created by Seminario method using MCPB.py
CysSG(135)-FE2-S3	52.84	115.18	Created by Seminario method using MCPB.py
CysSG(135)-FE2-S4	75.62	109.23	Created by Seminario method using MCPB.py
CysSG(135)-FE2-S1	47.43	105.58	Created by Seminario method using MCPB.py
S1-FE2-S3	62.28	106.66	Created by Seminario method using MCPB.py
S1-FE2-S4	34.47	110.31	Created by Seminario method using MCPB.py
S1-FE3-S2	16.26	111.70	Created by Seminario method using MCPB.py
S1-FE3-S4	22.23	110.45	Created by Seminario method using MCPB.py
S1-FE4-S2	28.28	112.69	Created by Seminario method using MCPB.py
S1-FE4-S3	59.25	106.50	Created by Seminario method using MCPB.py
GnOE(155)-FE3-S2	34.09	115.71	Created by Seminario method using MCPB.py
GnOE(155)-FE3-S4	59.86	109.31	Created by Seminario method using MCPB.py
GnOE(155)-FE3-S1	51.72	107.58	Created by Seminario method using MCPB.py
CysSG(129)-FE4-S2	53.37	112.42	Created by Seminario method using MCPB.py
CysSG(129)-FE4-S3	46.08	111.58	Created by Seminario method using MCPB.py
CysSG(129)-FE4-S1	64.85	104.01	Created by Seminario method using MCPB.py
CysSG(139)-FE5-S6	53.22	109.82	Created by Seminario method using MCPB.py
CysSG(139)-FE5-S7	45.83	111.05	Created by Seminario method using MCPB.py
CysSG(139)-FE5-S8	51.92	109.60	Created by Seminario method using MCPB.py
S6-FE5-S7	28.91	110.49	Created by Seminario method using MCPB.py
S6-FE5-S8	36.64	105.86	Created by Seminario method using MCPB.py
S6-FE7-S8	42.99	106.90	Created by Seminario method using MCPB.py
S6-FE8-S7	20.07	108.94	Created by Seminario method using MCPB.py
S7-FE5-S8	59.08	109.89	Created by Seminario method using MCPB.py
S7-FE6-S8	37.10	109.86	Created by Seminario method using MCPB.py
CysSG(86)-FE6-S7	45.25	109.29	Created by Seminario method using MCPB.py
CysSG(86)-FE6-S8	63.88	116.82	Created by Seminario method using MCPB.py
CysSG(86)-FE6-S5	53.36	107.61	Created by Seminario method using MCPB.py
S5-FE6-S7	29.55	104.82	Created by Seminario method using MCPB.py
S5-FE6-S8	44.49	107.73	Created by Seminario method using MCPB.py
S5-FE7-S6	29.29	111.70	Created by Seminario method using MCPB.py
S5-FE7-S8	76.82	108.93	Created by Seminario method using MCPB.py
S5-FE8-S6	42.92	109.34	Created by Seminario method using MCPB.py
S5-FE8-S7	16.77	103.08	Created by Seminario method using MCPB.py
CysSG(78)-FE7-S6	62.02	113.79	Created by Seminario method using MCPB.py
CysSG(78)-FE7-S8	55.31	113.14	Created by Seminario method using MCPB.py
CysSG(78)-FE7-S5	62.04	102.35	Created by Seminario method using MCPB.py
CysSG(81)-FE8-S6	47.02	116.54	Created by Seminario method using MCPB.py
CysSG(81)-FE8-S7	51.70	110.21	Created by Seminario method using MCPB.py
CysSG(81)-FE8-S5	53.95	107.88	Created by Seminario method using MCPB.py
2C-C -GnOE(155)	80.0	120.40	
CX-CT-CysSG(90)	50.0	108.60	AA cys (was CT-CT-SH)
CX-CT-CysSG(135)	50.0	108.60	AA cys (was CT-CT-SH)
CX-CT-CysSG(129)	50.0	108.60	AA cys (was CT-CT-SH)
CX-CT-CysSG(139)	50.0	108.60	AA cys (was CT-CT-SH)
CX-CT-CysSG(86)	50.0	108.60	AA cys (was CT-CT-SH)
CX-CT-CysSG(78)	50.0	108.60	AA cys (was CT-CT-SH)
CX-CT-CysSG(81)	50.0	108.60	AA cys (was CT-CT-SH)

N -C -GnOE(155)	80.0	122.90	AA general	
CysSG(90)-CT-H1	50.0	109.50	AA cyx	changed based on NMA nmodes
CysSG(135)-CT-H1	50.0	109.50	AA cyx	changed based on NMA nmodes
CysSG(129)-CT-H1	50.0	109.50	AA cyx	changed based on NMA nmodes
CysSG(139)-CT-H1	50.0	109.50	AA cyx	changed based on NMA nmodes
CysSG(86)-CT-H1	50.0	109.50	AA cyx	changed based on NMA nmodes
CysSG(78)-CT-H1	50.0	109.50	AA cyx	changed based on NMA nmodes
CysSG(81)-CT-H1	50.0	109.50	AA cyx	changed based on NMA nmodes

DIHE

2C-2C-C -GnOE(155)	1	0.0	0.0	1.0	
2C-C -GnOE(155)-FE3	3	0.00	0.00	3.0	Treat as zero by MCPB.py
C -GnOE(155)-FE3-S2	3	0.00	0.00	3.0	Treat as zero by MCPB.py
C -GnOE(155)-FE3-S4	3	0.00	0.00	3.0	Treat as zero by MCPB.py
C -GnOE(155)-FE3-S1	3	0.00	0.00	3.0	Treat as zero by MCPB.py
CT-CysSG(90)-FE1-S2	3	0.00	0.00	3.0	Treat as zero by MCPB.py
CT-CysSG(90)-FE1-S3	3	0.00	0.00	3.0	Treat as zero by MCPB.py
CT-CysSG(90)-FE1-S4	3	0.00	0.00	3.0	Treat as zero by MCPB.py
CT-CysSG(135)-FE2-S3	3	0.00	0.00	3.0	Treat as zero by MCPB.py
CT-CysSG(135)-FE2-S4	3	0.00	0.00	3.0	Treat as zero by MCPB.py
CT-CysSG(135)-FE2-S1	3	0.00	0.00	3.0	Treat as zero by MCPB.py
CT-CysSG(129)-FE4-S2	3	0.00	0.00	3.0	Treat as zero by MCPB.py
CT-CysSG(129)-FE4-S3	3	0.00	0.00	3.0	Treat as zero by MCPB.py
CT-CysSG(129)-FE4-S1	3	0.00	0.00	3.0	Treat as zero by MCPB.py
CT-CysSG(139)-FE5-S6	3	0.00	0.00	3.0	Treat as zero by MCPB.py
CT-CysSG(139)-FE5-S7	3	0.00	0.00	3.0	Treat as zero by MCPB.py
CT-CysSG(139)-FE5-S8	3	0.00	0.00	3.0	Treat as zero by MCPB.py
CT-CysSG(86)-FE6-S7	3	0.00	0.00	3.0	Treat as zero by MCPB.py
CT-CysSG(86)-FE6-S8	3	0.00	0.00	3.0	Treat as zero by MCPB.py
CT-CysSG(86)-FE6-S5	3	0.00	0.00	3.0	Treat as zero by MCPB.py
CT-CysSG(78)-FE7-S6	3	0.00	0.00	3.0	Treat as zero by MCPB.py
CT-CysSG(78)-FE7-S8	3	0.00	0.00	3.0	Treat as zero by MCPB.py
CT-CysSG(78)-FE7-S5	3	0.00	0.00	3.0	Treat as zero by MCPB.py
CT-CysSG(81)-FE8-S6	3	0.00	0.00	3.0	Treat as zero by MCPB.py
CT-CysSG(81)-FE8-S7	3	0.00	0.00	3.0	Treat as zero by MCPB.py
CT-CysSG(81)-FE8-S5	3	0.00	0.00	3.0	Treat as zero by MCPB.py
CX-CT-CysSG(90)-FE1	3	0.00	0.00	3.0	Treat as zero by MCPB.py
CX-CT-CysSG(135)-FE2	3	0.00	0.00	3.0	Treat as zero by MCPB.py
CX-CT-CysSG(129)-FE4	3	0.00	0.00	3.0	Treat as zero by MCPB.py
CX-CT-CysSG(139)-FE5	3	0.00	0.00	3.0	Treat as zero by MCPB.py
CX-CT-CysSG(86)-FE6	3	0.00	0.00	3.0	Treat as zero by MCPB.py
CX-CT-CysSG(78)-FE7	3	0.00	0.00	3.0	Treat as zero by MCPB.py
CX-CT-CysSG(81)-FE8	3	0.00	0.00	3.0	Treat as zero by MCPB.py
H -N -C -GnOE(155)	1	2.5	180.0	-2.0	JCC,7,(1986),230
H -N -C -GnOE(155)	1	2.0	0.0	1.0	J.C.cistrans-NMA DE
FE1-CysSG(90)-CT-H1	3	0.00	0.00	3.0	Treat as zero by MCPB.py
FE2-S3-FE1-S4	3	0.00	0.00	3.0	Treat as zero by MCPB.py
FE2-CysSG(135)-CT-H1	3	0.00	0.00	3.0	Treat as zero by MCPB.py
FE3-S2-FE1-S3	3	0.00	0.00	3.0	Treat as zero by MCPB.py
FE3-S2-FE1-S4	3	0.00	0.00	3.0	Treat as zero by MCPB.py
FE3-S1-FE2-S3	3	0.00	0.00	3.0	Treat as zero by MCPB.py
FE3-S1-FE2-S4	3	0.00	0.00	3.0	Treat as zero by MCPB.py
FE4-S2-FE1-S3	3	0.00	0.00	3.0	Treat as zero by MCPB.py
FE4-S2-FE1-S4	3	0.00	0.00	3.0	Treat as zero by MCPB.py
FE4-S2-FE3-S4	3	0.00	0.00	3.0	Treat as zero by MCPB.py

FE4-S3-FE1-S4	3	0.00	0.00	3.0	Treat as zero by MCPB.py
FE4-S3-FE2-S4	3	0.00	0.00	3.0	Treat as zero by MCPB.py
FE4-S1-FE2-S3	3	0.00	0.00	3.0	Treat as zero by MCPB.py
FE4-S1-FE2-S4	3	0.00	0.00	3.0	Treat as zero by MCPB.py
FE4-S1-FE3-S2	3	0.00	0.00	3.0	Treat as zero by MCPB.py
FE4-S1-FE3-S4	3	0.00	0.00	3.0	Treat as zero by MCPB.py
FE4-CysSG(129)-CT-H1	3	0.00	0.00	3.0	Treat as zero by MCPB.py
FE5-CysSG(139)-CT-H1	3	0.00	0.00	3.0	Treat as zero by MCPB.py
FE6-S7-FE5-S8	3	0.00	0.00	3.0	Treat as zero by MCPB.py
FE6-CysSG(86)-CT-H1	3	0.00	0.00	3.0	Treat as zero by MCPB.py
FE7-S6-FE5-S7	3	0.00	0.00	3.0	Treat as zero by MCPB.py
FE7-S6-FE5-S8	3	0.00	0.00	3.0	Treat as zero by MCPB.py
FE7-S5-FE6-S7	3	0.00	0.00	3.0	Treat as zero by MCPB.py
FE7-S5-FE6-S8	3	0.00	0.00	3.0	Treat as zero by MCPB.py
FE7-CysSG(78)-CT-H1	3	0.00	0.00	3.0	Treat as zero by MCPB.py
FE8-S6-FE5-S7	3	0.00	0.00	3.0	Treat as zero by MCPB.py
FE8-S6-FE5-S8	3	0.00	0.00	3.0	Treat as zero by MCPB.py
FE8-S6-FE7-S8	3	0.00	0.00	3.0	Treat as zero by MCPB.py
FE8-S7-FE5-S8	3	0.00	0.00	3.0	Treat as zero by MCPB.py
FE8-S7-FE6-S8	3	0.00	0.00	3.0	Treat as zero by MCPB.py
FE8-S5-FE6-S7	3	0.00	0.00	3.0	Treat as zero by MCPB.py
FE8-S5-FE6-S8	3	0.00	0.00	3.0	Treat as zero by MCPB.py
FE8-S5-FE7-S6	3	0.00	0.00	3.0	Treat as zero by MCPB.py
FE8-S5-FE7-S8	3	0.00	0.00	3.0	Treat as zero by MCPB.py
FE8-CysSG(81)-CT-H1	3	0.00	0.00	3.0	Treat as zero by MCPB.py
N -C -GnOE(155)-FE3	3	0.00	0.00	3.0	Treat as zero by MCPB.py
CysSG(90)-FE1-S2-FE3	3	0.00	0.00	3.0	Treat as zero by MCPB.py
CysSG(90)-FE1-S2-FE4	3	0.00	0.00	3.0	Treat as zero by MCPB.py
CysSG(90)-FE1-S3-FE2	3	0.00	0.00	3.0	Treat as zero by MCPB.py
CysSG(90)-FE1-S3-FE4	3	0.00	0.00	3.0	Treat as zero by MCPB.py
CysSG(90)-FE1-S4-FE2	3	0.00	0.00	3.0	Treat as zero by MCPB.py
CysSG(90)-FE1-S4-FE3	3	0.00	0.00	3.0	Treat as zero by MCPB.py
S2-FE1-S3-FE2	3	0.00	0.00	3.0	Treat as zero by MCPB.py
S2-FE1-S3-FE4	3	0.00	0.00	3.0	Treat as zero by MCPB.py
S2-FE1-S4-FE2	3	0.00	0.00	3.0	Treat as zero by MCPB.py
S2-FE1-S4-FE3	3	0.00	0.00	3.0	Treat as zero by MCPB.py
S2-FE3-S4-FE1	3	0.00	0.00	3.0	Treat as zero by MCPB.py
S2-FE3-S4-FE2	3	0.00	0.00	3.0	Treat as zero by MCPB.py
S2-FE3-S1-FE2	3	0.00	0.00	3.0	Treat as zero by MCPB.py
S2-FE4-S3-FE1	3	0.00	0.00	3.0	Treat as zero by MCPB.py
S2-FE4-S3-FE2	3	0.00	0.00	3.0	Treat as zero by MCPB.py
S2-FE4-S1-FE2	3	0.00	0.00	3.0	Treat as zero by MCPB.py
S2-FE4-S1-FE3	3	0.00	0.00	3.0	Treat as zero by MCPB.py
S3-FE1-S4-FE2	3	0.00	0.00	3.0	Treat as zero by MCPB.py
S3-FE1-S4-FE3	3	0.00	0.00	3.0	Treat as zero by MCPB.py
S3-FE2-S4-FE1	3	0.00	0.00	3.0	Treat as zero by MCPB.py
S3-FE2-S4-FE3	3	0.00	0.00	3.0	Treat as zero by MCPB.py
S3-FE4-S2-FE1	3	0.00	0.00	3.0	Treat as zero by MCPB.py
S3-FE4-S2-FE3	3	0.00	0.00	3.0	Treat as zero by MCPB.py
S3-FE4-S1-FE2	3	0.00	0.00	3.0	Treat as zero by MCPB.py
S3-FE4-S1-FE3	3	0.00	0.00	3.0	Treat as zero by MCPB.py
S4-FE2-S3-FE1	3	0.00	0.00	3.0	Treat as zero by MCPB.py
S4-FE3-S2-FE1	3	0.00	0.00	3.0	Treat as zero by MCPB.py
S4-FE3-S1-FE2	3	0.00	0.00	3.0	Treat as zero by MCPB.py
CysSG(135)-FE2-S3-FE1	3	0.00	0.00	3.0	Treat as zero by MCPB.py

CysSG(135)-FE2-S3-FE4	3	0.00	0.00	3.0	Treat as zero by MCPB.py
CysSG(135)-FE2-S4-FE1	3	0.00	0.00	3.0	Treat as zero by MCPB.py
CysSG(135)-FE2-S4-FE3	3	0.00	0.00	3.0	Treat as zero by MCPB.py
CysSG(135)-FE2-S1-FE3	3	0.00	0.00	3.0	Treat as zero by MCPB.py
CysSG(135)-FE2-S1-FE4	3	0.00	0.00	3.0	Treat as zero by MCPB.py
S1-FE2-S3-FE1	3	0.00	0.00	3.0	Treat as zero by MCPB.py
S1-FE2-S3-FE4	3	0.00	0.00	3.0	Treat as zero by MCPB.py
S1-FE2-S4-FE1	3	0.00	0.00	3.0	Treat as zero by MCPB.py
S1-FE2-S4-FE3	3	0.00	0.00	3.0	Treat as zero by MCPB.py
S1-FE3-S2-FE1	3	0.00	0.00	3.0	Treat as zero by MCPB.py
S1-FE3-S2-FE4	3	0.00	0.00	3.0	Treat as zero by MCPB.py
S1-FE3-S4-FE1	3	0.00	0.00	3.0	Treat as zero by MCPB.py
S1-FE3-S4-FE2	3	0.00	0.00	3.0	Treat as zero by MCPB.py
S1-FE4-S2-FE1	3	0.00	0.00	3.0	Treat as zero by MCPB.py
S1-FE4-S2-FE3	3	0.00	0.00	3.0	Treat as zero by MCPB.py
S1-FE4-S3-FE1	3	0.00	0.00	3.0	Treat as zero by MCPB.py
S1-FE4-S3-FE2	3	0.00	0.00	3.0	Treat as zero by MCPB.py
GnOE(155)-C -2C-HC	1	0.08	180.0	-3.0	Junmei et al, 1999 (HC-CT-C -O)
GnOE(155)-C -2C-HC	1	0.0	0.0	-2.0	
GnOE(155)-C -2C-HC	1	0.8	0.0	1.0	
GnOE(155)-FE3-S2-FE1	3	0.00	0.00	3.0	Treat as zero by MCPB.py
GnOE(155)-FE3-S2-FE4	3	0.00	0.00	3.0	Treat as zero by MCPB.py
GnOE(155)-FE3-S4-FE1	3	0.00	0.00	3.0	Treat as zero by MCPB.py
GnOE(155)-FE3-S4-FE2	3	0.00	0.00	3.0	Treat as zero by MCPB.py
GnOE(155)-FE3-S1-FE2	3	0.00	0.00	3.0	Treat as zero by MCPB.py
GnOE(155)-FE3-S1-FE4	3	0.00	0.00	3.0	Treat as zero by MCPB.py
CysSG(129)-FE4-S2-FE1	3	0.00	0.00	3.0	Treat as zero by MCPB.py
CysSG(129)-FE4-S2-FE3	3	0.00	0.00	3.0	Treat as zero by MCPB.py
CysSG(129)-FE4-S3-FE1	3	0.00	0.00	3.0	Treat as zero by MCPB.py
CysSG(129)-FE4-S3-FE2	3	0.00	0.00	3.0	Treat as zero by MCPB.py
CysSG(129)-FE4-S1-FE2	3	0.00	0.00	3.0	Treat as zero by MCPB.py
CysSG(129)-FE4-S1-FE3	3	0.00	0.00	3.0	Treat as zero by MCPB.py
CysSG(139)-FE5-S6-FE7	3	0.00	0.00	3.0	Treat as zero by MCPB.py
CysSG(139)-FE5-S6-FE8	3	0.00	0.00	3.0	Treat as zero by MCPB.py
CysSG(139)-FE5-S7-FE6	3	0.00	0.00	3.0	Treat as zero by MCPB.py
CysSG(139)-FE5-S7-FE8	3	0.00	0.00	3.0	Treat as zero by MCPB.py
CysSG(139)-FE5-S8-FE6	3	0.00	0.00	3.0	Treat as zero by MCPB.py
CysSG(139)-FE5-S8-FE7	3	0.00	0.00	3.0	Treat as zero by MCPB.py
S6-FE5-S7-FE6	3	0.00	0.00	3.0	Treat as zero by MCPB.py
S6-FE5-S7-FE8	3	0.00	0.00	3.0	Treat as zero by MCPB.py
S6-FE5-S8-FE6	3	0.00	0.00	3.0	Treat as zero by MCPB.py
S6-FE5-S8-FE7	3	0.00	0.00	3.0	Treat as zero by MCPB.py
S6-FE7-S8-FE5	3	0.00	0.00	3.0	Treat as zero by MCPB.py
S6-FE7-S8-FE6	3	0.00	0.00	3.0	Treat as zero by MCPB.py
S6-FE7-S5-FE6	3	0.00	0.00	3.0	Treat as zero by MCPB.py
S6-FE8-S7-FE5	3	0.00	0.00	3.0	Treat as zero by MCPB.py
S6-FE8-S7-FE6	3	0.00	0.00	3.0	Treat as zero by MCPB.py
S6-FE8-S5-FE6	3	0.00	0.00	3.0	Treat as zero by MCPB.py
S6-FE8-S5-FE7	3	0.00	0.00	3.0	Treat as zero by MCPB.py
S7-FE5-S8-FE6	3	0.00	0.00	3.0	Treat as zero by MCPB.py
S7-FE5-S8-FE7	3	0.00	0.00	3.0	Treat as zero by MCPB.py
S7-FE6-S8-FE5	3	0.00	0.00	3.0	Treat as zero by MCPB.py
S7-FE6-S8-FE7	3	0.00	0.00	3.0	Treat as zero by MCPB.py
S7-FE8-S6-FE5	3	0.00	0.00	3.0	Treat as zero by MCPB.py
S7-FE8-S6-FE7	3	0.00	0.00	3.0	Treat as zero by MCPB.py

S7-FE8-S5-FE6	3	0.00	0.00	3.0	Treat as zero by MCPB.py
S7-FE8-S5-FE7	3	0.00	0.00	3.0	Treat as zero by MCPB.py
S8-FE6-S7-FE5	3	0.00	0.00	3.0	Treat as zero by MCPB.py
S8-FE7-S6-FE5	3	0.00	0.00	3.0	Treat as zero by MCPB.py
S8-FE7-S5-FE6	3	0.00	0.00	3.0	Treat as zero by MCPB.py
CysSG(86)-FE6-S7-FE5	3	0.00	0.00	3.0	Treat as zero by MCPB.py
CysSG(86)-FE6-S7-FE8	3	0.00	0.00	3.0	Treat as zero by MCPB.py
CysSG(86)-FE6-S8-FE5	3	0.00	0.00	3.0	Treat as zero by MCPB.py
CysSG(86)-FE6-S8-FE7	3	0.00	0.00	3.0	Treat as zero by MCPB.py
CysSG(86)-FE6-S5-FE7	3	0.00	0.00	3.0	Treat as zero by MCPB.py
CysSG(86)-FE6-S5-FE8	3	0.00	0.00	3.0	Treat as zero by MCPB.py
S5-FE6-S7-FE5	3	0.00	0.00	3.0	Treat as zero by MCPB.py
S5-FE6-S7-FE8	3	0.00	0.00	3.0	Treat as zero by MCPB.py
S5-FE6-S8-FE5	3	0.00	0.00	3.0	Treat as zero by MCPB.py
S5-FE6-S8-FE7	3	0.00	0.00	3.0	Treat as zero by MCPB.py
S5-FE7-S6-FE5	3	0.00	0.00	3.0	Treat as zero by MCPB.py
S5-FE7-S6-FE8	3	0.00	0.00	3.0	Treat as zero by MCPB.py
S5-FE7-S8-FE5	3	0.00	0.00	3.0	Treat as zero by MCPB.py
S5-FE7-S8-FE6	3	0.00	0.00	3.0	Treat as zero by MCPB.py
S5-FE8-S6-FE5	3	0.00	0.00	3.0	Treat as zero by MCPB.py
S5-FE8-S6-FE7	3	0.00	0.00	3.0	Treat as zero by MCPB.py
S5-FE8-S7-FE5	3	0.00	0.00	3.0	Treat as zero by MCPB.py
S5-FE8-S7-FE6	3	0.00	0.00	3.0	Treat as zero by MCPB.py
CysSG(78)-FE7-S6-FE5	3	0.00	0.00	3.0	Treat as zero by MCPB.py
CysSG(78)-FE7-S6-FE8	3	0.00	0.00	3.0	Treat as zero by MCPB.py
CysSG(78)-FE7-S8-FE5	3	0.00	0.00	3.0	Treat as zero by MCPB.py
CysSG(78)-FE7-S8-FE6	3	0.00	0.00	3.0	Treat as zero by MCPB.py
CysSG(78)-FE7-S5-FE6	3	0.00	0.00	3.0	Treat as zero by MCPB.py
CysSG(78)-FE7-S5-FE8	3	0.00	0.00	3.0	Treat as zero by MCPB.py
CysSG(81)-FE8-S6-FE5	3	0.00	0.00	3.0	Treat as zero by MCPB.py
CysSG(81)-FE8-S6-FE7	3	0.00	0.00	3.0	Treat as zero by MCPB.py
CysSG(81)-FE8-S7-FE5	3	0.00	0.00	3.0	Treat as zero by MCPB.py
CysSG(81)-FE8-S7-FE6	3	0.00	0.00	3.0	Treat as zero by MCPB.py
CysSG(81)-FE8-S5-FE6	3	0.00	0.00	3.0	Treat as zero by MCPB.py
CysSG(81)-FE8-S5-FE7	3	0.00	0.00	3.0	Treat as zero by MCPB.py

IMPR

X -X -C -GnOE(155)	10.5	180.	2.	JCC,7,(1986),230

NONB

FE1	1.4090	0.0172100000	IOD set for Fe2+ ion from Li et al. JCTC, 2013, 9, 2733
FE2	1.4090	0.0172100000	IOD set for Fe2+ ion from Li et al. JCTC, 2013, 9, 2733
FE3	1.4090	0.0172100000	IOD set for Fe2+ ion from Li et al. JCTC, 2013, 9, 2733
FE4	1.4090	0.0172100000	IOD set for Fe2+ ion from Li et al. JCTC, 2013, 9, 2733
FE5	1.4090	0.0172100000	IOD set for Fe2+ ion from Li et al. JCTC, 2013, 9, 2733
FE6	1.4090	0.0172100000	IOD set for Fe2+ ion from Li et al. JCTC, 2013, 9, 2733
FE7	1.4090	0.0172100000	IOD set for Fe2+ ion from Li et al. JCTC, 2013, 9, 2733
FE8	1.4090	0.0172100000	IOD set for Fe2+ ion from Li et al. JCTC, 2013, 9, 2733
CysSG(78)	2.0000	0.2500	W. Cornell CH3SH and CH3SCH3 FEP's
CysSG(81)	2.0000	0.2500	W. Cornell CH3SH and CH3SCH3 FEP's
CysSG(86)	2.0000	0.2500	W. Cornell CH3SH and CH3SCH3 FEP's
CysSG(90)	2.0000	0.2500	W. Cornell CH3SH and CH3SCH3 FEP's
CysSG(129)	2.0000	0.2500	W. Cornell CH3SH and CH3SCH3 FEP's
CysSG(135)	2.0000	0.2500	W. Cornell CH3SH and CH3SCH3 FEP's
CysSG(139)	2.0000	0.2500	W. Cornell CH3SH and CH3SCH3 FEP's

GnOE(155)	1.6612	0.2100	OPLS
S1	2.0000	0.2500	S ion
S2	2.0000	0.2500	S ion
S3	2.0000	0.2500	S ion
S4	2.0000	0.2500	S ion
S5	2.0000	0.2500	S ion
S6	2.0000	0.2500	S ion
S7	2.0000	0.2500	S ion
S8	2.0000	0.2500	S ion

Table S4. A representation of human DPD parameters and coordinate files for Model 2 (LSDA/LANL2DZ): AMBER_VFFDT parameter file

#This file is generated by the VFFDT program for AMBER.

MASS			
S3	32.06500	2.90000	; S
F3	55.84500	2.05000	; Fe

BOND	(kcal/mol/Å)	(Å)	
S3-F3	89.23755	2.22545	; Averaged bond related parameters from VFFDT
F3-F3	59.53185	2.43332	; Averaged bond related parameters from VFFDT
SG-F3	39.7711	2.3294	; Averaged manually with SD(10.7658) and SD(1.0804)
O-F3	24.9728	1.9340	; Averaged manually with SD(3.5472) and SD(0.9834)

ANGL	(kcal mol/rad)	(degrees)	
S3-F3-S3	39.52095	109.20	; Averaged angle related parameters from VFFDT
S3-F3-F3	43.33559	73.06	; Averaged angle related parameters from VFFDT
F3-S3-F3	26.86171	66.28	; Averaged angle related parameters from VFFDT
F3-F3-F3	52.40760	60.00	; Averaged angle related parameters from VFFDT
SG-F3-F3	38.8905	145.0629	; Averaged manually with SD(11.4418) and SD(9.6176)
SG-F3-S3	36.1370	113.2785	; Averaged manually with SD(6.7190) and SD(9.1432)
H-SG-F3	23.5646	104.3280	; Averaged manually with SD(4.0419) and SD(8.1811)
F3-O-C	33.8107	134.0050	; Averaged manually with SD(4.1504) and SD(8.2116)
S3-F3-O	41.2264	115.3200	; Averaged manually with SD(5.1145) and SD(8.6921)
O-F3-F3	56.4637	150.9850	; Averaged manually with SD(5.3204) and SD(8.7809)
F3-SG-C	60.7114	112.8094	; Averaged manually with SD(15.3253) and SD(9.2780)
F3-SG-HS	0.0000	0.0000	; Not calculated
F3-SG-2C	0.0000	0.0000	; Not calculated
SG-F3-SG	0.0000	0.0000	; Not calculated
O-F3-SG	0.0000	0.0000	; Not calculated

DIHE					
F3-S3-F3-S3	1	0.00000	0.00	1.00	; Please check it manually.
S3-F3-S3-F3	1	0.00000	0.00	1.00	; Please check it manually.
S3-F3-F3-S3	1	0.00000	0.00	1.00	; Please check it manually.
S3-F3-F3-F3	1	0.00000	0.00	1.00	; Please check it manually.
F3-S3-F3-F3	1	0.00000	0.00	1.00	; Please check it manually.
F3-F3-S3-F3	1	0.00000	0.00	1.00	; Please check it manually.
F3-F3-F3-F3	1	0.00000	0.00	1.00	; Please check it manually.

```
F3-F3-F3-SG     1  0.00000   0.00  1.00       ; Please check it manually.
F3-F3-F3-O      1  0.00000   0.00  1.00       ; Please check it manually.
F3-F3-SG-2C     1  0.00000   0.00  1.00       ; Please check it manually.
F3-S3-F3-O      1  0.00000   0.00  1.00       ; Please check it manually.
F3-F3-SG-HS     1  0.00000   0.00  1.00       ; Please check it manually.
F3-S3-F3-SG     1  0.00000   0.00  1.00       ; Please check it manually.
F3-F3-O-C       1  0.00000   0.00  1.00       ; Please check it manually.
S3-F3-SG-HS     1  0.00000   0.00  1.00       ; Please check it manually.
S3-F3-F3-SG     1  0.00000   0.00  1.00       ; Please check it manually.
S3-F3-F3-O      1  0.00000   0.00  1.00       ; Please check it manually.
S3-F3-SG-2C     1  0.00000   0.00  1.00       ; Please check it manually.
S3-F3-O-C       1  0.00000   0.00  1.00       ; Please check it manually.
SG-F3-F3-O      1  0.00000   0.00  1.00       ; Please check it manually.
SG-F3-F3-SG     1  0.00000   0.00  1.00       ; Please check it manually.
HS-SG-F3-SG     1  0.00000   0.00  1.00       ; Please check it manually.
SG-F3-SG-2C     1  0.00000   0.00  1.00       ; Please check it manually.
O-F3-SG-2C      1  0.00000   0.00  1.00       ; Please check it manually.
O-F3-SG-HS      1  0.00000   0.00  1.00       ; Please check it manually.
C-O-F3-SG       1  0.00000   0.00  1.00       ; Please check it manually.


IMPR

NONB
  F3       1.4090  0.0172100000      IOD set for Fe2+ ion from Li et al. JCTC, 2013, 9, 2733
  S3       2.0000  0.2500         W. Cornell CH3SH and CH3SCH3 FEP's
```

Table S5. Listing of charge allocation to all the atoms interacting with the metal center (B3LYP/6-31G*)

A) Model 1

Atom	Atomic charge		Atom	Atomic charge		Atom	Atomic
FE1	0.489		S1	- 0.802		GLN(156)	- 0.467
FE2	0.725		S2	- 0.841		CYS(136)	- 0.726
FE3	0.622		S3	- 0.866		CYS(130)	- 0.570
FE4	0.614		S4	- 0.822		CYS(91)	- 0.672
FE5	0.553		S5	- 0.758		CYS(82)	- 0.752
FE6	0.705		S6	- 1.025		CYS(87)	- 0.823
FE7	0.667		S7	- 0.799		CYS(140)	- 0.718
FE8	0.638		S8	- 0.752		CYS(79)	- 0.722

Table S6. Comparison of **A** bond length, **B** internal and **C** external angles (Å) calculated with X-ray, DFT (B3LYP), (LSDA/LANL2DZ) and GFN1-xTB: extended TB method for the molecular cluster model ([$Fe^{2+}_4S^{2-}_4$ (S-Cys)$_3$(S-Gln)]) 1026A of Native DPD protein.

A

**Geometry**	**Bond length** *(Å)*						
**Model System**	**$Fe^{2+}_4S^{2-}_4$(S-Cys)$_3$(O-Gln): 1026A Clusters**						
**Bond**	**X-ray**	**[1] QM ([2] DFT)**				**AFTER [3] MD**	
**Bond description**	**1H7X**	**[4] B3LYP (Model 1)**		**[5] LSDA/LANL2DZ (Model 2)**		**Model 1**	**Model 2**
	**Bond length** *(Å)*	**Equilibrium bond length [req]** *(Å)*	**Force constant [Kr]** ***(kcal mol^{-1} Å^{-2})***	**Equilibrium bond length [req]** *(Å)*	**Force constant [Kr]** *(kcal mol^{-1} Å^{-2})*	**Bond length** *(Å)* **Mean & [6] SD**	**Bond length** *(Å)* **Mean & [6] SD**
FE1-S2	2.51	2.28	56.10	2.22	89.23	2.28±0.16	2.22±0.21
FE1-S3	2.42	2.20	55.10	2.22	89.23	2.20±0.17	2.23±0.14
FE1-S4	2.58	2.22	66.10	2.22	89.23	2.24± 0.24	2.23± 0.25
FE1-SG91	2.32	2.39	44.60	2.33	39.77	2.36±0.03	2.35±0.02
FE2-S1	2.61	2.26	54.70	2.22	89.23	2.28±0.23	2.23±0.27
FE2-S3	2.64	2.18	66.50	2.22	89.23	2.21±0.30	2.22±0.30
FE2-S4	2.73	2.25	62.00	2.22	89.23	2.27±0.33	2.22±0.43
FE2-SG136	2.37	2.33	53.40	2.33	39.77	2.36±0.01	2.34±0.02
FE3-S1	2.43	2.26	49.30	2.22	89.23	2.25±0.13	2.23±0.14
FE3-S2	2.40	2.30	57.30	2.22	89.23	2.30±0.07	2.23±0.12
FE3-S4	2.56	2.24	76.30	2.22	89.23	2.23±0.23	2.23±0.23
FE3-OE156	1.89	1.92	60.40	1.93	24.97	1.98±0.06	1.92±0.02
FE4-S1	2.60	2.25	50.50	2.22	89.23	2.24±0.25	2.23±0.26
FE4-S2	2.60	2.29	54.00	2.22	89.23	2.25±0.25	2.24±0.25
FE4-S3	2.37	2.19	55.60	2.22	89.23	2.18±0.13	2.22±0.11
FE4-SG130	2.36	2.40	36.40	2.33	39.77	2.38±0.01	2.34±0.01

[1] QM: quantum mechanics, [2] DFT: density functional theory, [3] MD: molecular dynamics, [4] B3LYP: Becke three-parameter hybrid exchange and Lee Yang Parr, [5] LSDA/LANL2DZ**:** Los Alamos double-zeta basis; [6] SD: standard deviation

B

Geometry	Internal Angle (⁰)						
**Model System**	**$Fe^{2+}{}_4S^{2-}{}_4$(S-Cys)₃(O-Gln): 1026A Clusters**						
**Angle**	**X-ray**	**[1] QM ([2] DFT)**				**AFTER [3] MD**	
**Angle description**	**1H7X**	**[4] B3LYP (Model 1)**		**[5] LSDA/LANL2DZ (Model 2)**		**Model 1**	**Model 2**
	**Average angle** *(⁰)*	**Average equilibrium Angle [Θ_{eq}]** *(⁰)*	**Force constant [KΘ]** *(kcal mol⁻¹rad⁻²)*	**Average equilibrium Angle [Θ_{eq}]** *(⁰)*	**Force constant [KΘ]** *(kcal mol⁻¹rad⁻²)*	**Angle (⁰) Mean & [6] SD**	**Angle (⁰) Mean & [6] SD**
FE1-S2-FE3	68.12	69.98	23.20	66.28	26.86	58.20±7.01	69.05±0.66
FE1-S2-FE4	65.87	65.65	53.67	66.28	26.86	64.32±0.29	69.61±2.64
FE1-S3-FE2	64.61	68.39	75.11	66.28	26.86	64.33±0.20	68.93±3.05
FE1-S3-FE4	65.86	68.59	29.62	66.28	26.86	67.08±0.86	67.81±1.37
FE1-S4-FE2	68.72	66.76	80.79	66.28	26.86	60.34±5.93	70.09±0.96
FE1-S4-FE3	67.60	72.04	26.76	66.28	26.86	63.80± 2.69	63.97± 2.56
FE2-S1-FE3	68.15	66.08	47.73	66.28	26.86	67.27±0.62	68.49±0.24
FE2-S3-FE4	71.15	68.33	61.07	66.28	26.86	65.35±4.10	69.07±1.47
FE2-S4-FE3	64.60	66.60	40.49	66.28	26.86	61.15±2.43	67.96±2.38
FE3-S1-FE4	71.92	65.300	53.87	66.28	26.86	62.67±6.54	67.05± 3.44
FE3-S2-FE4	67.59	64.15	20.95	66.28	26.86	58.20±6.64	68.10±0.36
FE4-S1-FE2	71.60	66.00	78.37	66.28	26.86	62.23±6.62	67.09±3.19
S1-FE2-S3	102.67	106.66	62.28	109.21	39.52	114.04±8.04	105.95±2.31
S1-FE2-S4	102.09	110.31	34.47	109.21	39.52	104.29± 1.55	105.23±2.22
S1-FE3-S2	107.87	111.70	16.26	109.21	39.52	105.68±1.54	105.77±1.48
S1-FE3-S4	107.47	110.45	22.23	109.21	39.52	104.41±2.16	108.21±0.52
S1-FE4-S2	113.03	112.69	28.28	109.21	39.52	111.05±1.40	110.56±1.40
S1-FE4-S3	101.97	106.50	59.25	109.21	39.52	105.86±2.75	109.33±5.20
S2-FE1-S3	107.61	109.45	43.74	109.21	39.52	112.63±3.55	105.74±1.32
S2-FE1-S4	102.08	103.16	18.56	109.21	39.52	111.25± 6.48	101.64±0.31
S2-FE3-S4	107.74	101.98	18.52	109.21	39.52	110.49± 1.94	109.93±1.55
S2-FE4-S3	106.71	109.42	60.52	109.21	39.52	108.75±1.44	108.36±1.17
S3-FE1-S4	107.71	109.98	35.95	109.21	39.52	112.97±3.71	107.41±0.21
S3-FE2-S4	105.38	109.74	69.35	109.21	39.52	109.61±2.99	105.77±0.27

[1] QM: quantum mechanics, [2] DFT: density functional theory, [3] MD: molecular dynamics, [4] B3LYP: Becke three-parameter hybrid exchange and Lee Yang Parr, [5] LSDA/LANL2DZ**:** Los Alamos double-zeta basis; [6] SD: standard deviation

C

Geometry	External Angle (⁰)						
Model System	$Fe_4S_4(S\text{-}Cys)_3(O\text{-}Gln)$: 1026A						
Bond calculations	X-ray	[1] QM ([2] DFT)				AFTER [3] MD	
	1H7X	[4] B3LYP (Model 1)		[5] GFN1-xTB (Model 2)		Model 1	Model 2
Bond description	Angle (*⁰*)	Equilibrium Angle (*⁰*)	Force constant (*kcal mol^{-1}rad^{-2}*)	Equilibrium Angle (*⁰*)	Force constant (*kcal mol^{-1}rad^{-2}*)	Angle (⁰) Mean & [6] SD	Angle (*⁰*) Mean & [6] SD
C -OE155-FE3	117.29	130.30	75.86	115.32	41.23	115.29± 1.41	114.42±2.02
CT-SG90-FE1	99.53	103.98	106.68	107.39	100.90	108.04±6.02	107.92±5.93
CT-SG135-FE2	102.34	107.39	100.90	107.40	100.90	105.06±1.92	107.41±3.58
CT-SG129-FE4	100.25	97.96	96.66	107.39	100.90	99.42±0.58	107.20±4.91
N-C-OE155-H	104.50	122.90	80.0	118.02	44.55	113.34±6.25	116.93±8.78
SG90-CT-H	107.85	109.50	50.0	104.33	23.56	100.25±5.37	103.85± 2.83
SG135-CT-H	108.36	109.50	50.0	104.33	23.56	109.45±0.77	103.96±3.11
SG129-CT-H	105.31	109.50	50.0	104.33	23.56	98.83±4.58	101.12±2.96
SG90-FE1-S2	106.08	111.30	41.30	113.28	36.14	108.03±1.38	112.68±4.66
SG90-FE1-S3	111.94	115.56	63.82	113.28	36.14	112.26±0.23	113.07±0.80
SG90-FE1-S4	116.45	106.49	65.80	113.28	36.14	106.80±6.82	113.79±1.88
SG135-FE2-S3	112.62	115.18	52.84	113.28	36.14	105.02±5.37	112.46±0.11
SG135-FE2-S4	107.32	109.23	75.62	113.28	36.14	105.13±1.54	113.63± 4.46
SG135-FE2-S1	100.34	105.58	47.43	113.28	36.14	108.76±5.95	113.69± 9.43
OE155-FE3-S2	109.87	111.71	34.09	113.08	40.55	109.75±0.08	111.78±1.35
OE155-FE3-S4	106.39	109.31	59.86	113.08	40.55	114.93±6.03	112.69±4.45
OE155-FE3-S1	105.27	107.58	51.72	113.08	40.55	108.63±2.37	111.81±4.62
SG129-FE4-S2	110.83	112.42	53.37	113.28	36.14	106.42±3.12	113.16±1.64
SG129-FE4-S3	113.52	111.58	46.08	113.28	36.14	110.62±2.05	113.01±0.36
SG129-FE4-S1	99.46	104.01	64.85	113.28	36.14	106.51±4.99	100.49±0.72

[1] QM: quantum mechanics, [2] DFT: density functional theory, [3] MD: molecular dynamics, [4] B3LYP: Becke three-parameter hybrid exchange and Lee Yang Parr, [5] GFN1-xTB: extended TB, [6] SD: standard deviation

Table S7. Comparison of **A** bond length, **B** internal and **C** external angles (Å) calculated with X-ray, DFT (B3LYP), (LSDA/LANL2DZ) and GFN1-xTB: extended TB method for the molecular cluster model ($[Fe^{2+}_4S^{2-}_4$ (S-Cys)$_3$(S-Gln)]) 1027A of Native DPD protein.

A

**Geometry**	**Bond length (Å)**						
**Model System**	**([Fe_4S_4(S-Cys)$_4$]): 1027A**						
**Bond calculations**	**X-ray**	**[1] QM ([2] DFT)**				**AFTER [3] MD**	
**Bond description**	**1H7X**	**[4] B3LYP (Model 1)**		**[5] LSDA/LANL2DZ (Model 2)**		**Model 1**	**Model 2**
	**Bond length** *(Å)*	**Equilibrium Bond length** *(Å)*	**Force constant [Kr]** ***(kcal mol^{-1} Å^{-2})***	**Equilibrium Bond length** *(Å)*	**Force constant [Kr]** ***(kcal mol^{-1} Å^{-2})***	**Bond length** *(Å)* **Mean & [6] SD**	**Bond length** *(Å)* **Mean & [6] SD**
FE1_S2	2.63	2.24	45.10	2.22	89.23	2.23±0.28	2.22±0.29
FE1_S3	2.43	2.25	64.30	2.22	89.23	2.24± 0.13	2.23±0.14
FE1_S4	2.60	2.21	58.30	2.22	89.23	2.23±0.26	2.23±0.26
FE1_SG140	2.30	2.40	36.10	2.33	39.77	2.39± 0.06	2.35±0.035
FE2_S1	2.61	2.24	66.30	2.22	89.23	2.25±0.25	2.22± 0.27
FE2_S3	2.55	2.24	69.00	2.22	89.23	2.23±0.22	2.22±0.23
FE2_S4	2.25	2.23	51.20	2.22	89.23	2.24±0.01	2.23±0,01
FE2_SG87	2.24	2.35	50.00	2.33	39.77	2.40±0.11	2.34±0.07
FE3_S1	2.44	2.24	60.80	2.22	89.23	2.24± 0.14	2.23±0.15
FE3_S2	2.38	2.23	45.30	2.22	89.23	2.32±0.04	2.23±0.11
FE3_S4	2.25	2.19	64.70	2.22	89.23	2.19± 0.13	2.23±0.11
FE3_SG79	2.30	2.37	40.20	2.33	39.77	2.40±0.07	2.35±0.04
FE4_S1	2.55	2.27	41.50	2.20	89.23	2.26±0.21	2.22±0.23
FE4_S2	2.25	2.27	61.80	2.22	89.23	2.28±0.02	2.23±0.01
FE4_S3	2.60	2.26	57.00	2.22	89.23	2.27± 0.23	2.22±0.27
FE4_SG82	2.40	2.41	37.10	2.33	39.77	2.37±0.02	2.33±0.05

[1] QM: quantum mechanics, [2] DFT: density functional theory, [3] MD: molecular dynamics, [4] B3LYP: Becke three-parameter hybrid exchange and Lee Yang Parr, [5] LSDA/LANL2DZ**:** Los Alamos double-zeta basis; [6] SD: standard deviation

B

**Geometry**	**Internal Angle (⁰)**						
**Model System**	**([Fe_4S_4(S-Cys)$_4$]): 1027A**						
**Bond calculations**	**X-ray**	**[1] QM ([2] DFT)**				**AFTER [3] MD**	
**Bond angle description**	**1H7X**	**[4] B3LYP (Model 1)**		**[5] LSDA/LANL2DZ (Model 2)**		**Model 1**	**Model 2**
	**Angle** *(⁰)*	**Equilibrium Angle** *(⁰)*	**Force constant** *(kcal $mol^{-1}rad^{-2}$)*	**Equilibrium Angle** *(⁰)*	**Force constant** *(kcal $mol^{-1}rad^{-2}$)*	**Angle** *(⁰)* **Mean & [6] SD**	**Angle** *(⁰)* **Mean & [6] SD**
FE1-S2-FE3	67.72	67.16	42.52	66.28	26.86	66.86±0.61	68.54±0.58
FE1-S2-FE4	67.87	67.08	61.00	66.28	26.86	65.27±1.84	68.40±0.12
FE1-S3-FE2	68.36	66.89	27.43	66.28	26.86	67.35±0.71	69.70±0.94
FE1-S3-FE4	64.95	70.89	36.63	66.28	26.86	63.30±1.17	67.45±1.77
FE1-S4-FE2	66.89	67.77	77.31	66.28	26.86	62.02±0.61	69.32±1.72
FE1-S4-FE3	70.03	67.02	46.02	66.28	26.86	60.89±6.46	67.74±1.62
FE2-S1-FE3	68.46	68.38	64.00	66.28	26.86	67.33±0.80	66.97±1.05
FE2-S3-FE4	72.01	67.59	35.00	66.28	26.86	64.79±5.11	67.77±3.00
FE2-S4-FE3	69.43	68.59	42.93	66.28	26.86	62.23±5.09	67.67±1.24
FE3-S1-FE4	67.82	66.06	54.32	66.28	26.86	64.75±2.17	68.51±0.49
FE3-S2-FE4	67.71	67.32	36.84	66.28	26.86	66.98±0.52	69.31±1.13
FE4-S1-FE2	69.42	66.62	67.61	66.28	26.86	62.78±4.07	67.45±1.40
S1-FE2-S3	107.69	104.82	49.55	109.21	39.52	108.31±0.43	109.66±1.39
S1-FE2-S4	104.28	107.73	29.29	109.21	39.52	107.45±2.25	103.33±0.67
S1-FE3-S2	117.43	111.70	44.49	109.21	39.52	117.93±0.35	109.85±5.36
S1-FE3-S4	107.59	108.93	76.82	109.21	39.52	109.23±1.16	109.66±1.46
S1-FE4-S2	106.71	109.34	42.92	109.21	39.52	108.31±1.13	110.03±2.35
S1-FE4-S3	101.60	103.08	16.77	109.21	39.52	112.49±7.70	105.50±2.76
S2-FE1-S3	107.70	110.60	28.91	109.21	39.52	109.76± 1.46	109.65±1.38
S2-FE1-S4	105.51	105.86	36.64	109.21	39.52	111.75±4.41	108.32±1.99
S2-FE3-S4	101.61	106.90	42.99	109.21	39.52	108.4±4.80	107.76±4.35
S2-FE4-S3	106.67	108.94	20.07	109.21	39.52	110.93±2.24	108.45±0.49
S3-FE1-S4	112.33	109.89	59.08	109.13	39.52	111.41±0.65	105.10±5.11

S3-FE2-S4	107.37	109.86	37.10	109.21	39.52	104.05±2.35	109.54±1.53

[1] QM: quantum mechanics, [2] DFT: density functional theory, [3] MD: molecular dynamics, [4] B3LYP: Becke three-parameter hybrid exchange and Lee Yang Parr, [5] LSDA/LANL2DZ: Los Alamos double-zeta basis; [6] SD: standard deviation

C

Geometry	External Angle (⁰)						
Model System	($[Fe_4S_4(S\text{-}Cys)_4]$): 1027A						
Bond calculations	X-ray	[1] QM ([2] DFT)				AFTER [3] MD	
Bond description	1H7X	[4] B3LYP (Model 1)		[5] GFN1-xTB (Model 2)		Model 1	Model 2
	Angle (⁰)	Equilibrium Angle (⁰)	Force constant *(kcal mol^{-1}rad^{-2})*	Equilibrium Angle (⁰)	Force constant *(kcal mol^{-1}rad^{-2})*	Angle (⁰) Mean & [6] SD	Angle (⁰) Mean & [6] SD
CT-SG78-FE3	123.43	108.65	100.02	115.32	41.23	108.76±10.37	114.21±6.52
CT-SG139-FE1	101.35	105.21	94.60	107.39	100.90	105.13±2.67	107.30±4.21
CT-SG86-FE2	102.43	107.61	107.61	107.39	100.90	110.52±6.43	107.14±4.04
CT-SG81-FE4	100.25	103.60	106.65	107.39	100.90	105.82±3.94	107.56±5.17
SG139-CT-H	114.50	122.90	80.0	118.02	44.55	114.93 ± 0.30	116.43±1.36
SG86-CT-H	107.58	109.50	50.0	104.33	23.56	106,63±0.67	100.91±4.71
SG78-CT-H	108.61	109.50	50.0	104.33	23.56	112.28±2.60	103.65±3.51
SG81-CT-H	105.01	109.50	50.0	104.33	23.56	110.72±4.03	103.12±1.34
SG139-FE1-S2	107.08	109.82	53.22	113.28	36.14	105.60±1.05	113.10±4.25
SG139-FE1-S3	115.94	111.05	45.22	113.28	36.14	110.80± 3.63	113.16±1.97
SG139-FE1-S4	112.45	109.60	51.92	113.28	36.14	112.52±0.05	112.64±0.13
SG86-FE2-S3	112.62	109.29	45.25	113.28	36.14	106.83±4.09	111.18±1.01
SG86-FE2-S4	117.32	116.82	63.88	113.28	36.14	112.53±3.38	113.37±2.80
SG86-FE2-S1	100.34	107.61	53.36	113.28	36.14	99.83±0.36	111.84±8.13
SG78-FE3-S2	109.78	113.79	62.02	113.28	36.14	110.21±0.30	112.49± 1.91
SG78-FE3-S4	106.39	113.14	55.31	113.28	36.14	109.30±2.05	112.38± 4.24
SG78-FE3-S1	105.27	102.35	62.04	113.28	36.14	108.77±2.47	113.18±5.59
SG81-FE4-S2	110.83	116.54	47.02	113.28	36.14	112.78±1.38	113.33±1.77
SG81-FE4-S3	113.26	110.21	51.70	113.28	36.14	107.39±4.15	112.98±0.20
SG81-FE4-S4	110.66	107.95	53.93	113.28	36.14	109.50±0.82	111.56±0.64

[1] QM: quantum mechanics, [2] DFT: density functional theory, [3] MD: molecular dynamics, [4] B3LYP: Becke three-parameter hybrid exchange and Lee Yang Parr, [5] GFN1-xTB: extended TB, [6] SD: standard deviation

Table S8. Dihedral related force constants for X-ray and post-MD simulation for both models' clusters ([Fe4S4(S-Cys)$_3$(S-Gln)]) and ([Fe_4S_4(S-Cys)$_4$]) of Native DPD protein

[1] QM: quantum mechanics, [2] DFT: density functional theory, [3] MD: molecular dynamics, [4] B3LYP: Becke three-parameter hybrid exchange and Lee Yang Parr, [5] GFN1-xTB: extended TB, [6] SD: standard deviation

Fe_4S_4 Clusters name	Geometry	Dihedral angles (⁰)						
	Model System	$Fe_4S_4(S\text{-}Cys)_3(O\text{-}Gln)$ and ($[Fe_4S_4(S\text{-}Cys)_4]$) Clusters						
	Bond	X-ray	[1] QM ([2] DFT)				AFTER [3] MD	
	Bond	X-ray	[4] B3LYP (Model 1)		[5] GFN1-xTB (Model 2)		Model 1	Model 2
	Description	Average dihedral angle (⁰)	Equilibrium Bond angle (⁰)	Force constant *(kcal/mol rad²)*	Equilibrium Bond angle (⁰)	Force constant (kcal/mol rad²)	Bond angle (⁰) Mean & [6] SD	Bond angle (⁰) Mean & [6] SD
**Cluster 1026_A**	S-FE-SG-CT	67.43					74.87± 5.26	76.64±6.51
	S-FE-OE-CT	58.67					62.56± 2.75	63.42±3.35
	CT-CT-FE-S	-112.78					-105.36±5.25	-105.79±4.94
	CT-CT-FE-SG	-54.79					-50.87±2.77	-49.93±3.43
**Cluster 1027_A**	S-FE-SG-CT	63.16					76.92±76.92	75.48± 8.71
	S-FE-SG-CT	56.23					63.54±5.17	63.74± 5.31
	CT-CT-FE-S	-111.87					-103.33±6.04	-105.67±4.38
	CT-CT-FE-SG	-56.42					-51.76±3.30	-51.93±3.1
**Cluster 1028_B**	S-FE-SG-CT	63.01					76.75±9.72	76.86±9.79
	S-FE-SG-CT	59.99					64.40±3.12	69.87±6.99
	CT-CT-FE-S	-112.61					-104.75±5.56	-105.23±5.22
	CT-CT-FE-SG	-55.28					-50.87±3.12	-50.11±3.66
**Cluster 1028_B**	S-FE-SG-CT	65.14					77.56±8.78	76.69±8.17
	S-FE-SG-CT	60.22					67.41±5.08	68.87±6.12
	CT-CT-FE-S	-110.94					-103.57± 5.21	-105.38± 3.93
	CT-CT-FE-SG	-58.23					-54.08±2.93	-52.39±4.13

Table S9. DPD *.pir* sequence file used for modeling human dihydropyramidine dehydrogenase structure based on pig crystal structure template and human target sequence

>P1;DPD_WT<br>sequence: DPD_WT: : : : :::0.00:0.00<br>MAPVLSKDSADIESILALNPRTQTHATLCSTSAKKLDKKHWKRNPDKNCFNCEKLENNFDDIKHTTL<br>GERGALREAMRCLKCADAPCQKSCPTNLDIKSFITSIANKNYYGAAKMIFSDNPLGLTCGMVCPTSDL<br>CVGGCNLYATEEGPINIGGLQQFAT<br>EVFKAMSIPQIRNPSLPPPEKMSEAYSAKIALFGAGPASISCASFLARLGYSDITIFEKQEYVGGLSTSEIP<br>QFRLPYDV<br>VNFEIELMKDLGVKIICGKSLSVNEMTLSTLKEKGYKAAFIGIGLPEPNKDAIFQGLTQDQGFYTSKDFL<br>PLVAKGSKAG<br>MCACHSPLPSIRGVVIVLGAGDTAFDCATSALRCGARRVFIVFRKGFVNIRAVPEEMELAKEEKCEFLP<br>FLSPRKVIVKG<br>GRIVAMQFVRTEQDETGKWNEDEDQMVHLKADVVISAFGSVLSDPKVKEALSPIKFNRWGLPEVDPE<br>TMQTSEAWVFAGG<br>DVVGLANTTVESVNDGKQASWYIHKYVQSQYGASVSAKPELPLFYTPIDLVDISVEMAGLKFINPFGL<br>ASATPATSTSMI<br>RRAFEAGWGFALTKTFSLDKDIVTNVSPRIIRGTTSGPMYGPGQSSFLNIELISEKTAAYWCQSVTELKA<br>DFPDNIVIAS<br>IMCSYNKNDWTELAKKSEDSGADALELNLSCPHGMGERGMGLACGQDPELVRNICRWVRQAVQIP<br>FFAKLTPNVTDIVSI<br>ARAAKEGGANGVTATNTVSGLMGLKSDGTPWPAVGIAKRTTYGGVSGTAIRPIALRAVTSIARALPG<br>FPILATGGIDSAE<br>SGLQFLHSGASVLQVCSAIQNQDFTVIEDYCTGLKALLYLKSIEELQDWDGQSPATVSHQKGKPVPRI<br>AELMDKKLPSFG<br>PYLEQRKKIIAENKIRLKEQNVAFSPLKRNCFIPKRPIPTIKDVIGKALQYLGTFGELSNVEQVVAMIDEE<br>MCINCGKCY<br>MTCNDSGYQAIQFDPETHLPTITDTCTGCTLCLSVCPIVDCIKMVSRTTPYEPKRGVPLSVNPVC.....*
>P1;1h7x_atm.pdb<br>structureX: 1h7x_atm.pdb::@::@:::0.00:0.00<br>-<br>APVLSKDVADIESILALNPRTQSHAALHSTLAKKLDKKHWKRNPDKNCFHCEKLENNFDDIKHTTLG<br>ERGALREAMRCL<br>KCADAPCQKSCPTHLDIKSFITSISNKNYYGAAKMIFSDNPLGLTCGMVCPTSDLCVGGCNLYATEEG<br>SINIGGLQQFAS<br>EVFKAMNIPQIRNPCLPSQEKMPEAYSAKIALLGAGPASISCASFLARLGYSDITIFEKQEYVGGLSTSEI<br>PQFRLPYDV<br>VNFEIELMKDLGVKIICGKSLSENEITLNTLKEEGYKAAFIGIGLPEPKTDDIFQGLTQDQGFYTSKDFLP<br>LVAKSSKAG<br>MCACHSPLPSIRGAVIVLGAGDTAFDCATSALRCGARRVFLVFRKGFVNIRAVPEEVELAKEEKCEFLP<br>FLSPRKVIVKG<br>GRIVAVQFVRTEQDETGKWNEDEDQIVHLKADVVISAFGSVLRDPKVKEALSPIKFNRWDLPEVDPET<br>MQTSEPWVFAGG<br>DIVGMANTTVESVNDGKQASWYIHKYIQAQYGASVSAKPELPLFYTPVDLVDISVEMAGLKFINPFGL<br>ASAAPTTSSSMI<br>RRAFEAGWGFALTKTFSLDKDIVTNVSPRIVRGTTSGPMYGPGQSSFLNIELISEKTAAYWCQSVTELK<br>ADFPDNIVIAS

IMCSYNKNDWMELSRKAEASGADALELNLSAPHGMGERGMGLACGQDPELVRNICRWVRQAVQIP
FFAKLTPNVTDIVSI
ARAAKEGGADGVTATNTVSGLMGLKADGTPWPAVGAGKRTTYGGVSGTAIRPIALRAVTTIARALP
GFPILATGGIDSAE
SGLQFLHSGASVLQVCSAVQNQDFTVIQDYCTGLKALLYLKSIEELQGWDGQSPGTESHQKGKPVPRI
AELMGKKLPNFG
PYLEQRKKIIAEEKMRLKEQNAAFPPLERKPFIPKKPIPAIKDVIGKALQYLGTFGELSNIEQVVAVIDEE
MCINCGKCY
MTCNDSGYQAIQFDPETHLPTVTDTCTGCTLCLSVCPIIDCIRMVSRTTPYEPKRGL--------.....*
